# The Use of Supported Acidic Ionic Liquids in Organic Synthesis

**DOI:** 10.3390/molecules19078840

**Published:** 2014-06-26

**Authors:** Rita Skoda-Földes

**Affiliations:** University of Pannonia, Institute of Chemistry, Department of Organic Chemistry, P. O. Box 158, Veszprém H-8201, Hungary; E-Mail: skodane@almos.uni-pannon.hu; Tel.: +36-88-624-719; Fax: +36-88-624-469

**Keywords:** ionic liquid, Lewis acidity, Brønsted acidity, supported, impregnation, grafting, sol-gel, acidic catalyst, recycling

## Abstract

Catalysts obtained by the immobilisation of acidic ionic liquids (ILs) on solid supports offer several advantages compared to the use of catalytically active ILs themselves. Immobilisation may result in an increase in the number of accessible active sites of the catalyst and a reduction of the amount of the IL required. The ionic liquid films on the carrier surfaces provide a homogeneous environment for catalytic reactions but the catalyst appears macroscopically as a dry solid, so it can simply be separated from the reaction mixture. As another advantage, it can easily be applied in a continuous fixed bed reactor. In the present review the main synthetic strategies towards the preparation of supported Lewis acidic and Brønsted acidic ILs are summarised. The most important characterisation methods and structural features of the supported ionic liquids are presented. Their efficiency in catalytic reactions is discussed with special emphasis on their recyclability.

## 1. Introduction

Acid-catalysed reactions involve one of the most important technologies applied in the chemical industry. Mineral acids, used in homogeneous phase reactions, usually show high catalytic activity, but they suffer from several drawbacks, such as the problems of side reactions, corrosion of the equipment and large amounts of acidic wastes. These reactions require tedious isolation of products, which can cause environmental problems.

Solid acids are more widely used since, as non-volatile materials, they are less harmful than traditional liquid acids [1]. However, these heterogeneous systems often show an inferior activity when compared to their homogeneous counterpart. The shortcomings include matrix-bound acidic sites, high molecular weight/active-site ratios, the necessity of using longer reaction times and rather extreme reaction conditions that may lead to deactivation because of coking.

**Figure 1:**
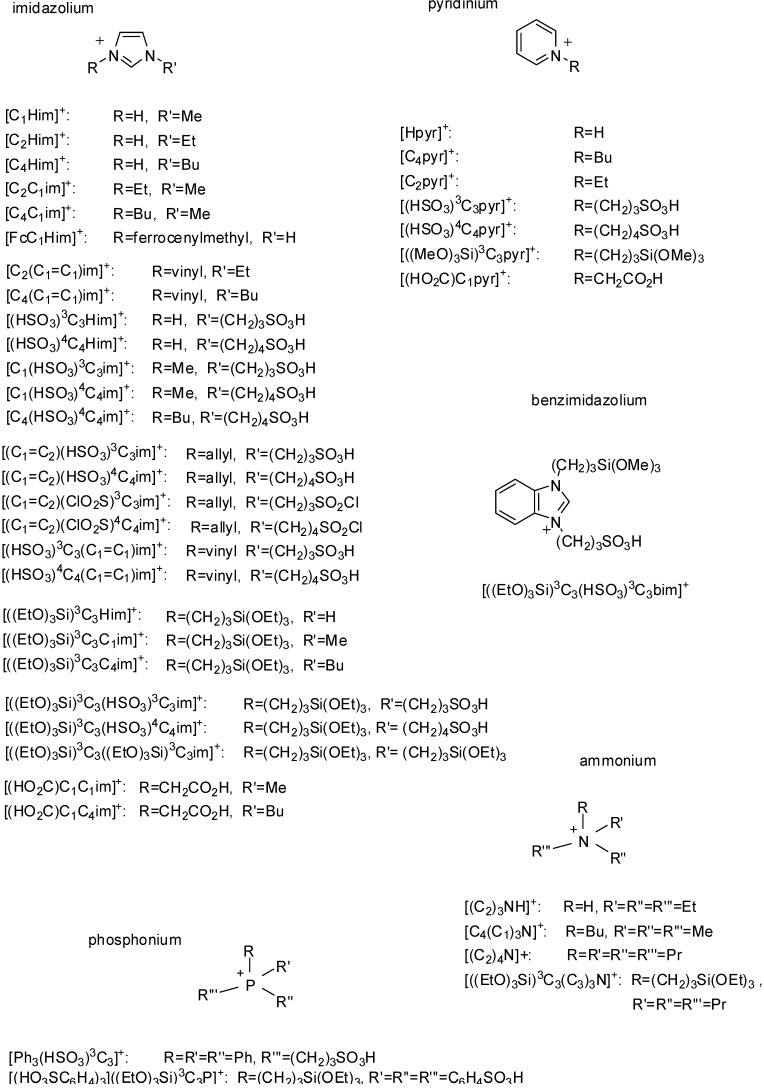
Cations of ionic liquids used during the synthesis of acidic SILPs.

As a result, great efforts were made towards the development of new catalysts. One possibility is the use of acidic ionic liquids (ILs). Synthesis, physicochemical properties and fields of application of these compounds have been summarised in several recent reviews [2,3,4,5,6]. ILs are salts consisting of bulky organic cations and inorganic or organic anions (Figure 1 shows the structures of cations of the ILs mentioned in this review). They melt at relatively low temperature, usually below 100 °C, have negligible vapour pressure and they are not flammable which makes them very easy and safe to handle. They dissolve both polar organic molecules and inorganic salts so they can replace volatile organic solvents. Mainly because of their low volatility, they are considered to be “green solvents”, however, their toxicity, investigated more thoroughly only recently, [7] should also be taken into account.

Because of the great variety of anion—cation pairs and the diversity in the side chains of the cations, an almost infinite IL combinations can be produced. Task-specific ILs can be developed by the fine-tune of their physical and chemical properties through a careful choice of the structure of the cation—anion pair. Such ionic liquids may have catalytic properties and can serve both as catalysts and solvents [8]. Besides, they make possible the immobilisation of organocatalysts [9] or organometallic catalysts [10], too. As most of the ILs do not dissolve apolar compounds, when the polarity of the products are sufficiently low, biphasic reactions may take place. After the completion of the reaction, the products can be separated by simple decantation and the IL phase can be reused.

Regarding catalytic properties, acidic ILs can be considered among the most important representatives of these compounds. Acidity may be due either to the anion or to the cation of the IL [5]. ILs with polynuclear metallic anions, such as chloroaluminate, chloroferrate or chlorozincate ions, show Lewis acidity when the Lewis acid (e.g., AlCl_3_), which forms the counteranion, is used in excess. In such acidic melts, the anions Al_2_Cl_7_^−^ and Al_3_Cl_10_^−^^−^ exist, which act as very strong Lewis acids.

The presence of the SO_2_Cl group in the side chain of the cation ([(C_1_=C_2_)(ClO_2_S)^4^C_4_im]^+^ or [(C_1_=C_2_)(ClO_2_S)^3^C_3_im]^+^, Figure 1) may also lead to Lewis acidity, since the LUMO orbital of methanesulfonyl chloride is mainly localised on sulfur atom and its energy is −2.35 eV as determined by a MOPAC-AM 1 calculation by Hagiwara *et al.* [11].

Brønsted acidic ILs can be prepared by the use of HSO_4_^−^ or H_2_PO_4_^−^ anions or by the introduction of alkane sulfonic acid or carboxylate acid groups as side chains of the cations. Combination of these two approaches leads to the so called “dual acidic” ionic liquids.

[C_1_Him][BF_4_] shows higher acidity compared to dialkylimidazolium ILs [12]. Its catalytic effect is mainly due to the more facile formation of a hydrogen bond between the C-2 hydrogen of the imidazolium ion and a suitable acceptor. Theoretical calculations proved that the presence of Brønsted acidic fragments on the cations could enhance the Lewis acidity on another site of the IL, e.g., at the C-2 position of the imidazolium ring [13,14]. At the same time, the counterions were also shown to have a decisive effect not only on the acidity of the IL but also on the location of the Lewis acidic site in the cation [14].

Despite such advantages as the potential in fine-tuning their structure in order to enhance selectivity and the possibility of their reuse, ILs also have some drawbacks. Because of the high viscosity of some ILs, their handling is often cumbersome and catalytic reactions are limited by diffusion processes. The use of ILs in their liquid form is inconvenient in an industrial continuous system, too. Biphasic IL—organic systems require large amounts of the expensive ILs, which also hinders industrial applications. These difficulties can be overcome by the use of supported ionic liquid phases (SILPs) prepared by the immobilisation of ILs on solid supports [15]. SILPs can also be helpful in the heterogenisation of transition metal catalysts [16,17] enabling an easy separation and recycling.

In the next sections, synthetic methods leading to supported acidic ionic liquids will be discussed and their structural properties will be summarised. Then their effect in various catalytic reactions will be surveyed, with special emphasis on their stability and recyclability.

## 2. Preparation and Characterization of Supported Acidic Ionic Liquids

SILPs are prepared by the deposition of ILs on the surface of a solid support with high surface area and suitable mechanical strength which is able to disperse them and consequently, to increase the number of accessible active sites of the catalyst. The lack of mass transfer restrictions leads to a much more efficient utilisation of the IL catalyst and allows a great reduction of the amount of the IL required. The ionic liquid films on the carrier surfaces provide a homogeneous environment for catalytic reactions but the SILP catalyst appears macroscopically as a dry solid. So the catalyst can be separated simply by filtration or decantation and can easily be applied in a continuous fixed bed reactor.

Both inorganic supports and organic polymers can be used as supports (see Table 1, Table 2 and Table 3). Inorganic supports usually have better thermal properties, while polymers offer the possibility to accommodate a higher loading of ILs. Among the inorganic supports, particularly alumina and silica have the advantages of low cost and ease of preparation. Compared to the amorphous silica gel, ordered mesoporous silica obtained by the condensation of tetraethoxysilane (TEOS) as silica source in the presence of a structure-directing agent, such as cetyl-trimethylammonium bromide, possesses excellent characteristics, e.g., stable mesoporous structure, high surface area, large pore volume and narrow pore distribution. Magnetic mesoporous materials combine the advantages of mesoporous and magnetic materials as carriers for ILs and simplify the recovery of the catalyst through magnetic separation. These supports are pretreated in most cases by heating or calcination to remove adsorbed water. In some examples, special pretreatment methods were applied that will be discussed in detail later. Further possibilities include the use of suitable synthetic polymers, or biopolymers, such as cellulose.

Immobilisation of ILs can be carried out by several methods: by impregnation of solid supports with the IL, by creating covalent bonds between the IL and the pre-formed support (grafting), by the confinement of the IL in the support material (obtained from a precursor of the support, e.g., tetraethoxysilane in the presence of the IL), or by copolymerisation of the IL with a suitable monomer.

Covalent bonds may be formed between the IL and the support even when the SILP is obtained by impregnation, e.g., in case of chloroaluminate ILs where anions are attached through the metal centre of the anion to a surface OH group [18]. Non-covalent interactions of the functional groups of anions, such as the SO_3_ group of [OTf]^−^, with solid surfaces were also detected by IR measurements [19].

As it will be discussed in the following sections, the success if immobilisation can be monitored by FT-IR or solid state ^13^C-, ^29^Si-, ^27^Al NMR measurements. The amount of IL can be calculated based on elemental analysis. Thermal properties of SILP are determined by TG-DTG analysis. SEM images give information about morphology and surface patterns of the particles, nanomaterials can be examined by TEM and the ordered structure of mesoporous material can be proved by XRD. BET surface (based on the Brunauer–Emmett–Teller (BET) theory), pore size and pore distribution are usually determined by N_2_ adsorption-desorption technique. Acidity can be calculated from the results of acid-base titration.

### 2.1. Immobilisation of Lewis Acidic ILs

Lewis acidic ILs are usually prepared by the addition of a sufficient amount of a metal halide (AlCl_3_, FeCl_3_, GaCl_3_, *etc.*) to an imidazolium halide. Immobilisation is achieved either by impregnation the support with the IL or with grafting the cation on the surface of the solid. The techniques used during immobilisation of Lewis acidic ILs, together with their use in catalytic reactions, are summarized in Table 1. 

**Table 1:** Preparation of supported Lewis acidic ILs and their application in catalytic reactions.

Ionic Liquid	Supporting Method	Support	Catalytic Reaction	Ref.
[C_4_C_1_im]Cl/AlCl_3_	impregnation	silica	alkylation	[20]
[C_4_C_1_im]Cl/AlCl_3_	impregnation	SiO_2_, Al_2_O_3_, TiO_2_, ZrO_2_	Friedel-Crafts alkylation	[21]
[C_4_C_1_im]Cl/AlCl_3_	impregnation	amorphous silica	Friedel-Crafts alkylation	[18]
[C_4_C_1_im]Cl/AlCl_3_	impregnation	glass, activated carbon	alkylation	[22]
[C_4_C_1_im]Cl/AlCl_3_	impregnation	silica gel, MCM-41, SBA-15	oligomerisation	[22]
[C_4_C_1_im]Cl/FeCl_3_	impregnation	amorphous silica, charcoal	Friedel-Crafts acylation	[23]
[(C_2_)_3_NH]Cl/AlCl_3_	impregnation	molecular sieves	epoxy ether cleavage	[24]
[(C_2_)_4_N][SnCl_5_]	impregnation	silica	Prins reaction	[25]
[C_2_C_1_im]Cl/AlCl_3_	impregnation	chemically pretreated silica gel	Friedel-Crafts alkylation	[26]
[C_4_C_1_im]Cl/AlCl_3_	impregnation	amorphous silica	alkylation	[27]
[((EtO)_3_Si)^3^C_3_C_1_im]Cl/AlCl_3_	grafting (condensation)	MCM-41	Friedel-Crafts alkylation	[18]
[((EtO)_3_Si)^3^C_3_C_1_im]Cl/AlCl_3_	grafting (condensation)	silica gel	alkylation	[22]
[((EtO)_3_Si)^3^C_3_C_1_im]Cl/AlCl_3_	grafting (condensation)	MCM-41	alkylation	[27]
[((EtO)_3_Si)^3^C_3_C_1_im]Cl/FeCl_3_	grafting (condensation)	MCM-41	Friedel-Crafts alkylation	[28]
[((MeO)_3_Si)^3^C_3_pyr][SnCl_5_] [((EtO)_3_Si)^3^C_3_(C_3_)_3_N][SnCl_5_]	grafting (condensation)	silica	Prins reaction	[25]
[((EtO)_3_Si)^3^C_3_((EtO)_3_Si)^3^C_3_im]Cl/InCl_3_	co-condensation with tetraethoxysilane	mesoporous silica	Friedel-Crafts alkylation	[29]
[(C_1_=C_2_)(ClO_2_S)^4^C_4_im][OTf] [(C_1_=C_2_)(ClO_2_S)^3^C_3_im][OTf]	grafting (radical chain transfer reaction)	3-mercaptopropylated silica	esterification, nitration	[30]
[(C_1_=C_2_)(ClO_2_S)^4^C_4_im][OTf]	grafting (radical chain transfer reaction)	3-mercaptopropylated silica	conjugate addition	[11]
[(C_1_=C_2_)(ClO_2_S)^4^C_4_im][OTf]	grafting (radical chain transfer reaction)	3-mercaptopropylated silica	domino Knoevenagel condensation/Michael addition	[31]
[C_1_Him]Cl/AlCl_3_	grafting (alkylation)	Merrifield resin	Knoevenagel condensation	[32]
[C_1_Him][FeCl_4_]	grafting (alkylation)	chloromethylated polystyrene	cycloaddition of epoxide or azridine with CO_2_	[33]
[Hpyr]Cl/AlCl_3_	grafting (alkylation)	Merrifield resin	Knoevenagel condensation	[34]
[C_4_(C_1_=C_1_)im][AlCl_4_] [C_2_(C_1_=C_1_)im][AlCl_4_] [C_2_pyr][AlCl_4_] [C_4_pyr][AlCl_4_]	polymerisation and alkylation	polymer	Diels-Alder reaction	[35]
[C_4_(C_1_=C_1_)im]Cl/GaCl_3_	copolymerization	polymer (styrene, [C_4_(C_1_=C_1_)im])	acetal formation	[36]
[C_4_(C_1_=C_1_)im][ZnCl_2_Br_2_] [C_2_(C_1_=C_1_)im][ZnCl_2_Br_2_]	polymerisation	polymer	cycloaddition of epoxide with CO_2_	[37]

#### 2.1.1. Immobilisation of Lewis Acidic ILs on Inorganic Supports

The use of silica supported Lewis acidic ILs, such as [C_4_C_1_im]Cl/AlCl_3_ and [Hpyr]Cl/AlCl_3_ was patented as early as 1993 by the co-workers of the Institut Francais du Petrole in an alkylation reaction [20]. The first careful examination of the catalysts obtained by the impregnation of various supports with chloroaluminate ILs was reported by Hölderich in 2000 [21]. Immobilisation was performed by a method of “incipient wetness” that means that the [C_4_C_1_im]Cl/AlCl_3_ ionic liquid was added to the support until the mixture lost the appearance of a dry powder. The excess was removed by extraction in a Soxhlet apparatus. The main change observed on the supports after impregnation was the decrease of the BET surface areas.

**Scheme 1:**
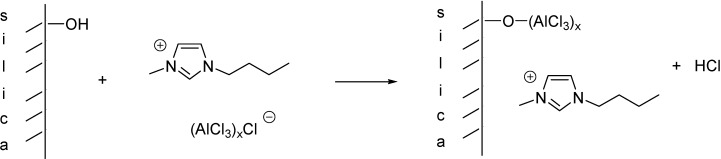
Supported Lewis acidic IL with a covalently bonded anion.

The wet impregnation method allowed the immobilisation of high amounts of chloroaluminate liquids on silica or alumina supports. Based on ^27^Al MAS NMR spectra, nearly none of the Al_2_Cl_7_^−^ dimers, typical for the pure ILs, remained and part of AlCl_3_ was believed to be covalently bound to the support through the M-OH groups of the latter (Scheme 1) [18]. This assumption was supported by FT-IR and ^29^Si CP MAS NMR spectroscopy that showed the disappearance of the hydroxyl groups on the surface of the supports. The amount of retained ILs for different supports after extraction changed in the order SiO_2_ (13 w%–35 w%) > Al_2_O_3_ (22 w%) > TiO_2_ (3 w%) > ZrO_2_. The low amount of immobilised IL in the latter two cases can be explained by the low surface area and lack of the M-OH groups in ZrO_2_ and TiO_2_ [21].

According to FT-IR spectra obtained after adsorption of pyridine showed the existence of both Lewis and Brønsted acid sites [22]. The IR peak at 1540 cm^−1^ involved a C–N^+^–H bending and was ascribed to the protonation of pyridine with Brønsted acid sites. The band at 1460 cm^−1^ was attributed to the adsorption of pyridine coordinated on Lewis acid sites, while the peak at 1490 cm^−1^ was associated to the vibration of the pyridine ring on both Brønsted and Lewis acid sites.

The ICP-AES analysis of these silica-supported IL catalysts before and after reaction showed the same Si/Al ratio and the reaction mixtures were free from aluminium that proved that no leaching took place [18].

In contrast, a strong leaching of the metal to the liquid phase was observed in reactions catalysed by analogous supported chloroferrate ILs [23]. It was assumed that no covalent bond was formed between the silica support and Fe, showing that the method is limited to anions which have a high affinity to oxygen.

At the same time, the deliberation of HCl in the immobilisation step led to a decomposition of structured supports, such as zeolite [21] or MCM-41 [18] showing close similarities to an amorphous material after impregnation.

Another possibility for immobilisation of ILs is impregnation of the solid support using a solution of the IL in an organic solvent. This method was used to deposit [(C_2_)_3_NH]Cl/AlCl_3_ on the surface of molecular sieves [24] and pyridinium chloride or tetrapropylammonium chloride on silica [25].

Upon interpreting catalytic data of Friedel-Crafts alkylation in the presence of a similarly prepared [C_2_C_1_im]Cl/AlCl_3_-silica catalyst, Wasserscheid and coworkers came to the conclusion that although the SILP system offered a more active catalytic surface, the Lewis acidity was lowered compared to neat chloroaluminate-organic biphasic systems due to the irreversible reaction of surface Si-OH groups with the acidic anions of the ionic liquid [26]. The acidity of SILP materials could be increased by higher ionic liquid loadings but this led to an increase in metal leaching during catalytic reactions. The problem could be overcome by a chemical pretreatment process that was carried out by stirring a mixture of the dichloromethane solution of the IL and the calcined support, followed by a washing procedure to remove all excess acid. Then this support was impregnated with a solution of the same IL and the solid catalyst was obtained by the evaporation of the solvent. Characterisation by temperature programmed desorption (TPD) of ammonia proved that pretreatment completely removed surface Si-OH groups from the support. The second impregnation step led to a catalyst with low Al leaching.

Decomposition of solid supports due to the formation of HCl [18,21] could be avoided by the preparation of SILP materials with covalently bonded cations.

**Scheme 2:**
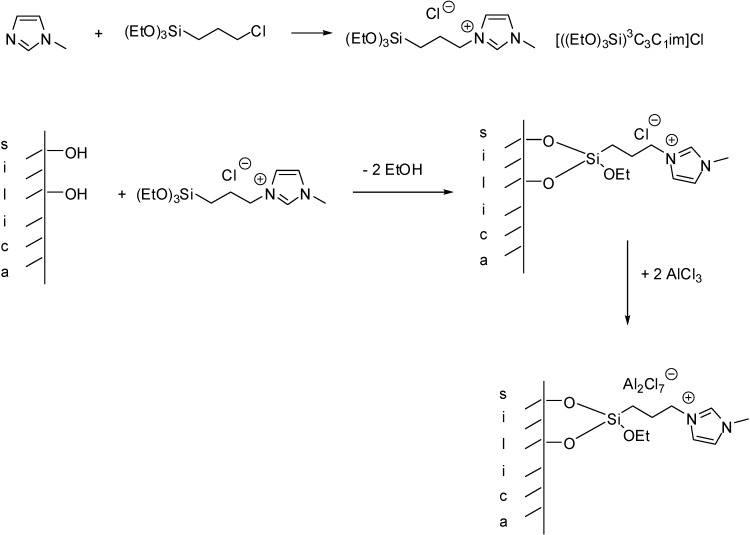
Immobilisation of a Lewis acidic IL via forming a covalent bond between the silica support and the cation.

The introduction of a triethoxysilyl group to the side chain of the imidazolium cation made it possible to attach the cation to the support via a condensation reaction between surface Si-OH groups of the support and Si-OEt groups of the IL. Addition of AlCl_3_ resulted in the formation of the Lewis acidic catalyst (Scheme 2) [18,27].

Two new peaks appearing at –54 ppm and –61 ppm in the ^29^Si CP MAS NMR spectrum, assigned to Si–O–SiR(OEt)_2_ and (Si-O)_2_–SiROEt groups, respectively, proved that the organic cation was grafted to the surface and the cations were bonded either via one or two Si–O–Si bonds. Upon addition of AlCl_3_, the ^27^Al CP MAS NMR spectrum of the resulting solid showed a peak at 102 ppm that was attributed to Al_2_Cl_7_^−^ ions and could be observed in pure ILs as well. The presence of some residual AlCl_3_ was also observed that could not be completely removed by Soxhlet extraction.

**Scheme 3:**
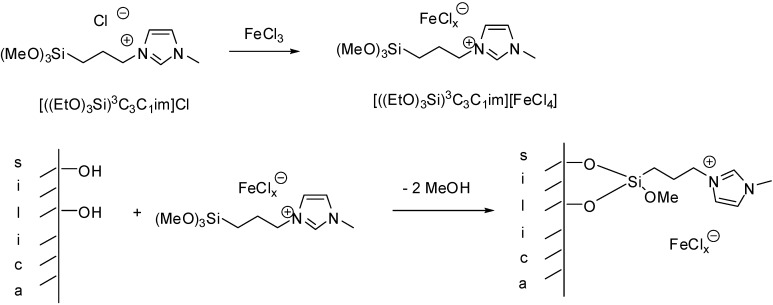
Preparation of Lewis acidic SILP material by carrying out ion exchange prior to the grafting step.

To avoid deposition of residual metal halide on the surface of the support, offering weak Lewis acidic sites, a slightly modified approach was used during the preparation of supported chloroferrate ILs, with the ion exchange step carried out before the grafting procedure (Scheme 3) [28].

According to XRD measurements, the MCM-41 support retained its structural integrity during the grafting process. N_2_ adsorption-desorption experiments showed decreased pore size, surface area and pore volume compared to the original support. Moreover, anchoring the ionic liquid moieties led to an increase of wall thickness.

**Scheme 4:**
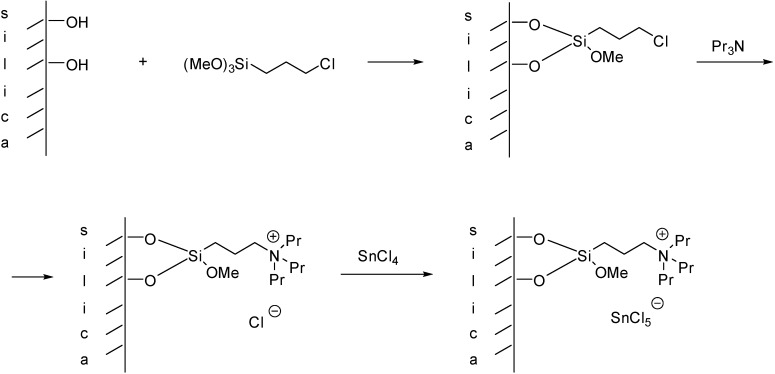
Immobilisation of a Lewis acidic IL by a stepwise procedure.

The acidic SILP material can also be prepared by a stepwise procedure, such as during the synthesis of a Lewis acidic tin catalyst depicted in Scheme 4 [25]. Interestingly, this catalyst was found to be more active in a Prins reaction than that obtained by grafting the pre-synthesised IL but no structural investigations were carried out to clarify this observation.

**Scheme 5:**
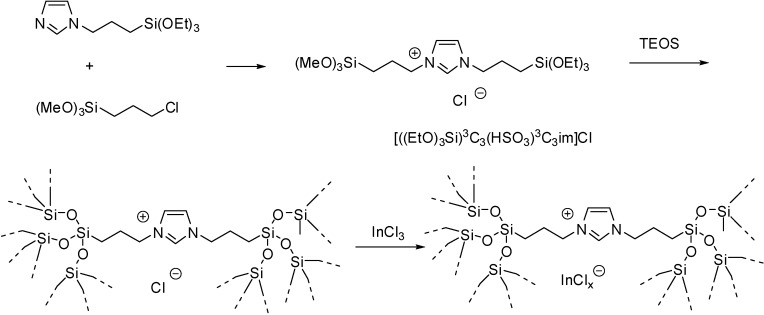
Synthesis of a Lewis acidic IL with a sol-gel method.

A periodic mesoporous organosilica with bridging imidazolium groups was obtained by the co-condensation of bis(trialkoxysilyl)alkyl imidazolium IL with tetraethoxysilane (TEOS) (Scheme 5) [29].

XRD measurements showed that the amount of the bridging organosilica precursor in the initial mixture had a great effect on the structural properties. The SILP material with a TEOS/IL precursor ratio of 18 possessed hexagonal pore system, although a less ordered one than SBA-15, but silica materials with a higher ratio of the imidazolium component had no mesoporous character. These observations were attributed to the different hydrolysis and/or condensation rates of organosilica precursors and TEOS and their different interaction with the template species. At the same time, in the first case TEM observations showed clearly the hexagonal arranged pore structure and nitrogen adsorption–desorption measurements proved the formation of large pore mesoporous materials with uniform cylindrical channels [29].

**Scheme 6:**
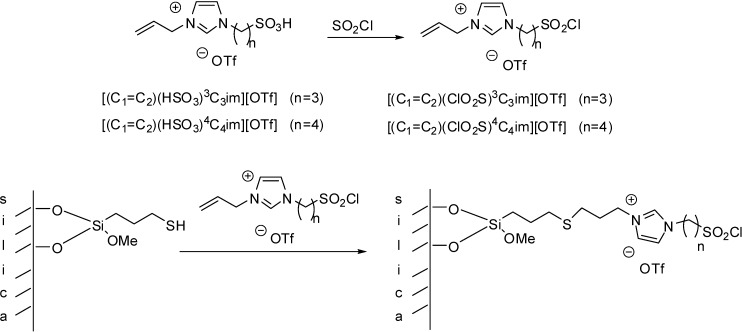
Conversion of Brønsted acidic [(C_1_=C_2_)(HSO_3_)^3^C_3_im][OTf] or [(C_1_=C_2_)(HSO_3_)^4^C_4_im][OTf] to the Lewis acidic sulfonyl chloride derivatives ([(C_1_=C_2_)(ClO_2_S)^3^C_3_im][OTf] or [(C_1_=C_2_)(ClO_2_S)^4^C_4_im][OTf]) and immobilisation on mercaptopropylated silica.

The Brønsted acidic ILs, [(C_1_=C_2_)(HSO_3_)^4^C_4_im][OTf] and [(C_1_=C_2_)(HSO_3_)^3^C_3_im][OTf] were converted to the Lewis acidic ILs with sulfonil chloride moiety ([(C_1_=C_2_)(ClO_2_S)^4^C_4_im][OTf] and [(C_1_=C_2_)(ClO_2_S)^4^C_4_im][OTf]) in the reaction with thionyl chloride (Scheme 6) [30].

The presence of the unsaturated side chain made it possible to form a covalent bond with mercaptopropylated silica by a radical chain transfer reaction [11,30,31]. The disappearance of the characteristic peaks of S–H at 2565 cm^−1^ and the allyl group at 1647 cm^−1^ in the FT-IR spectra, as well as the lack of the carbon signals typical for an allyl group in the solid state ^13^C-NMR proved the formation of the thioether bond [30].

#### 2.1.2. Immobilisation of Lewis Acidic ILs on Organic Supports

Instead of silica and related materials, polymers can also be used for the immobilisation of Lewis acidic ILs. Functionalised polymers can be obtained by the modification of a suitable polymer or by the polymerization of IL monomers with unsaturated side chains.

**Scheme 7:**
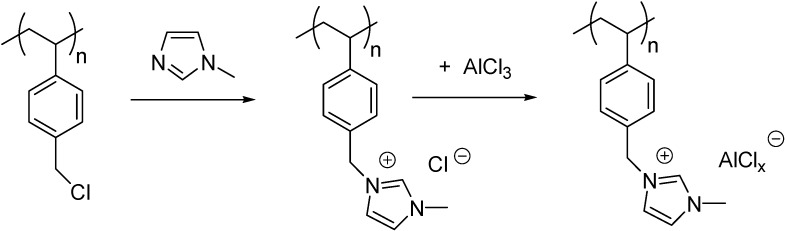
Immobilisation of an imidazolium IL on a Merrifield resin.

Imidazolium or pyridinium cations were obtained by the alkylation of 1-methylimidazole (Scheme 7) [32,33] or pyridine [34] with a Merrifield resin and then were converted to the acidic catalysts by a subsequent reaction with AlCl_3_ [32,34] or FeCl_3_ [33].

These catalysts were found to be stable to air and moisture retaining their activity for more than a year [32]. This stability was attributed to the hydrophobic nature of polystyrene that was able to protect the water-sensitive Lewis acid from hydrolysis by atmospheric moisture [34].

The formation of the covalent bonds was supported by FT-IR spectra of the functionalised resin that showed the appearance of new peaks at 575, 500, 430 cm^−1^ in chloroaluminate SIPLs, assigned to Al–Cl stretching modes of the Al_2_Cl_7_^−^ anion [32]. In case of the chloroferrate IL, the disappearance of the bands at 1263 and 671 cm^−1^ of the CH_2_Cl, together with the presence of four typical peaks of the imidazolium cation (1606, 1566, 1154, and 1085 cm^−1^), proved that alkylation was carried out successfully [33]. In the latter case a considerable change in the surface of the functionalised polymer was observed on SEM pictures, showing that the surface became rough in comparison with that of the support.

**Scheme 8:**
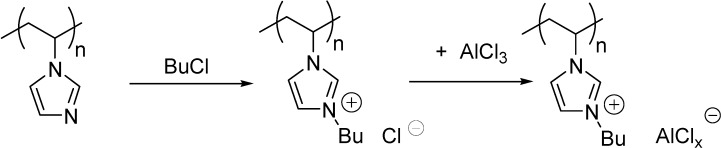
Preparation of a chloroaluminate IL on a poly(1-vinylimidazole) support.

Alkylation of polymers obtained by the polymerisation of 4-vinylpyridine or 1-vinylimidazole (Scheme 8) [35] or by the copolymerisation of 1-vinylimidazole and styrene [36] served as another approach for the incorporation of IL cations into the polymeric support. 

The thermal behaviour of the Lewis acidic catalyst depicted in Scheme 8 was found to be quite different from that of the imidazolium chloride precursor, implying that chloroaluminate anion was tightly coupled with the cations [35].

**Scheme 9:**
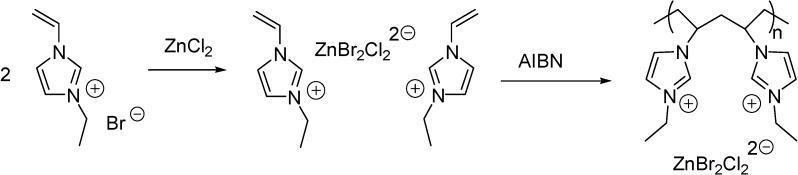
Polymerisation of zinc tetrahalide ILs.

At the same time, because of problems encountered during separation of product and starting material in the synthesis of a polymeric zinc tetrahalide IL, a direct polymerisation of the Lewis acidic IL was found to be a more useful method, than the addition of ZnCl_2_ to the polymer (Scheme 9) [37].

### 2.2. Immobilisation of Brønsted Acidic ILs

Immobilisation techniques used for Brønsted acidic ILs are summarised in Table 2.

**Table 2:** Preparation of supported Brønsted acidic ILs and their application in catalytic reactions.

Ionic Liquid	Supporting Method	Support	Catalytic Reaction	Ref.
[NH_4_][H_2_PO_4_]	impregnation	alumina	multicomponent condensation	[38]
[(HSO_3_)^4^C_4_pyr][HSO_4_]	impregnation	silica (Aerosil 300)	cyclocondensation	[39]
[(HSO_3_)^4^C_4_pyr][HSO_4_]	impregnation	silica (Aerosil 300)	aldol condensation	[40]
[C_1_(HSO_3_)^4^C_4_im][OTf] [C_4_(HSO_3_)^4^C_4_im][OTf] [C_1_(HSO_3_)^4^C_4_im][HSO_4_] [C_4_(HSO_3_)^4^C_4_im][HSO_4_]	impregnation	silica gel	oligomerisation	[41]
[C_1_(HSO_3_)^4^C_4_im][OTf]	impregnation	silica gel	oligomerisation	[42]
[C_1_(HSO_3_)^3^C_3_im][OTf]	ion exchange	montmorillonite clay	transesterifcation	[43]
[(HO_2_C)C_1_C_1_im][BF_4_] [(HO_2_C)C_1_C_1_im]Cl [(HO_2_C)C_1_pyr]Cl	sol-gel	silica	deoximation	[44]
[((EtO)_3_Si)^3^C_3_C_1_im][HSO_4_]	grafting (condensation)	silica material (obtained from TEOS)	Baeyer-Villiger oxidation	[45]
[((EtO)_3_Si)^3^C_3_C_1_im][HSO_4_]	grafting (condensation)	silica material (obtained from TEOS)	multicomponent condensation	[46,47,48,49,50]
[((EtO)_3_Si)^3^C_3_C_1_im][HSO_4_]	grafting (condensation)	magnetic nanoparticles	multicomponent condensation (Biginelli reaction)	[51]
[((EtO)_3_Si)^3^C_3_(HSO_3_)^3^C_3_im]Cl	grafting (condensation)	silica	hydrolysis	[52]
[((EtO)_3_Si)^3^C_3_(HSO_3_)^3^C_3_bim]Cl	grafting (condensation)	silica gel	transesterification	[53]
[((EtO)_3_Si)^3^C_3_(HSO_3_)^3^C_3_bim]Cl	grafting (condensation)	silica gel	multicomponent condensation	[54]
[((EtO)_3_Si)^3^C_3_(HSO_3_)^4^C_4_im][HSO_4_]	grafting (condensation)	silica gel	multicomponent condensation	[55]
[((EtO)_3_Si)^3^C_3_(HSO_3_)^4^C_4_im][HSO_4_]	grafting (condensation)	nano-silica (Cabosil 20)	multicomponent condensation	[56]
[((EtO)_3_Si)^3^C_3_(HSO_3_)^4^C_4_im][HSO_4_]	grafting (condensation)	magnetic nanoparticles coated with silica	multicomponent condensation	[57,58]
[((HO_3_S)C_6_H_4_)_3_((EtO)_3_Si)^3^C_3_P]Cl	grafting (condensation)	magnetic nanoparticles	acetal formation	[59]
[(C_1_=C_2_)(HSO_3_)^4^C_4_im][OTf] [(C_1_=C_2_)(HSO_3_)^3^C_3_im][OTf]	grafting (radical chain transfer reaction)	3-mercaptopropylated silica	esterification, nitration	[30]
[(C_1_=C_2_)(HSO_3_)^4^C_4_im][OTf]	grafting (radical chain transfer reaction)	3-mercaptopropylated silica	conjugate addition	[11]
[(C_1_=C_2_)(HSO_3_)^4^C_4_im][OTf]	grafting (radical chain transfer reaction)	3-mercaptopropylated silica	domino Knoevenagel condensation/Michael addition	[31]
[(HSO_3_)^3^C_3_(C_1_=C_1_)im][HSO_4_]	grafting (radical chain transfer reaction)	3-mercaptopropylated silica	esterification	[60]
[(C_1_=C_2_)(HSO_3_)^4^C_4_im][OTf]	grafting (radical chain transfer reaction)	3-mercaptopropylated magnetic mesoporous silica	esterification	[61]
[((EtO)_3_Si)^3^C_3_(HSO_3_)^3^C_3_im][HSO_4_]	sol-gel method	silica (obtained from TEOS)	acetal formation	[62]
[FcC_1_Him][HSO_4_]	grafting (alkylation)	Merrifield resin	allylation of aldehydes	[63]
[(HSO_3_)^3^C_3_Him][HSO_4_]	grafting (alkylation)	Merrifield resin	esterification	[64]
[(HSO_3_)^3^C_3_Him][ HSO_4_]	grafting (alkylation)	chloromethylated polystyrene	nitration	[65]
[(HSO_3_)^3^C_3_Him][HSO_4_]	grafting (alkylation)	silica polystyrene hybrid	esterification	[66]
[(HSO_3_)^4^C_4_pyr][HSO_4_]	grafting alkylation	poly(4-vinylpyridine)	domino Knoevenagel condensation/Michael reaction	[67]
[(HSO_3_)^4^C_4_pyr][HSO_4_]	grafting alkylation	poly(4-vinylpyridine)	multicomponent reaction	[68]
[[(HSO_3_)^4^C_4_melamine][HSO_4_]	grafting (alkylation)	melamine-formaldehyde resin	acetal formation	[69]
[(HSO_3_)^3^C_3_pyr]/[PW_12_O_40_]	polymerisation	poly(4-vinylpyridine)	cyclocondensation	[70]
[(HSO_3_)^4^C_4_(C_1_=C_1_)im][OTf]	copolymerisation	polymer (styrene, [(HSO_3_)^4^C_4_(C_1_=C_1_)im])	acetal formation	[71]
[(HSO_3_)^4^C_4_(C_1_=C_1_)im][HSO_4_]	copolymerisation	polymer	muticomponent condensation	[72]
[((EtO)_3_Si)^3^C_3_C_4_im][HSO_4_]	grafting (condensation)	cellulose	multicomponent condensation	[73]

#### 2.2.1. Immobilisation of Brønsted Acidic ILs on Inorganic Supports

Brønsted acidic ILs ([NH_4_][H_2_PO_4_] [38] [(HSO_3_)^4^C_4_pyr][HSO_4_] [39,40], [C_1_(HSO_3_)^4^C_4_im][HSO_4_], [C_1_(HSO_3_)^4^C_4_im][OTf], [C_4_(HSO_3_)^4^C_4_im][HSO_4_], [C_4_(HSO_3_)^4^C_4_im][OTf] [41,42] were immobilised on solid supports such as silica [39,40,41,42] or alumina [38] by impregnation with a solution of the IL. The solid catalysts were obtained after evaporation of the solvent or by filtration of the impregnated support. Immobilisation of the IL was proved by a decrease in the BET surface area and acidity could be determined by acid-base titrations [40] or NH_3_ chemisorption [42].

Both mesoporous and microporous silica materials were used as solid supports for the immobilisation of [C_1_(HSO_3_)^4^C_4_im][OTf] [42]. As it could be expected, immobilisation of the IL led to a loss of the BET surface of the supports. From the experimental data of the nitrogen adsorption/desorption isotherms of the supports and the solid catalysts, it could be concluded that the pores of mesoporous supports retained their shape during the catalyst preparation process, although the total pore volume values decreased because of the active IL film on the wall of the pores. On the other hand, the microporous supports contained narrow shaped pores that became almost totally filled with IL film during the catalyst preparation.

A sulfonic acid functionalised IL ([C_1_(HSO_3_)^4^C_4_im][OTf]) was introduced into clay interlayers by ion exchange [43]. Immobilisation was established by the electrostatic interactions between the negatively charged interlayers and the positively charged cation.

Supported acidic ILs were also obtained by a sol-gel method, *i.e.*, by the confinement of imidazolium and pyridinium ILs with carboxylic acid side chains (such as [(HO_2_C)C_1_C_1_im]Cl, [(HO_2_C)C_1_C_4_im]Cl and [(HO_2_C)C_1_pyr]Cl) in silica matrix [44]. Tetraethoxysilane was hydrolysed in the presence of the ILs to obtain the solid catalyst.

Although non-covalent attachment of the IL to the support bears some indisputable advantages, such as increased flexibility in the choice of both of the support material and IL component, non-covalent attachment may enhance leaching of the catalyst from the support, leading to higher losses in activity.

Supports, such as silica, magnetic nanoparticles, silica-coated nanoparticles or polymers were treated with the appropriately functionalised cations of the ionic liquids to obtain the heterogeneous catalysts.

As it was already shown during the preparation of Lewis acidic SILPs, in case of silica materials condensation between surface OH groups and trialkoxysilyl groups of the side chains of the cations leads to the formation of the covalent bonds.

**Scheme 10:**
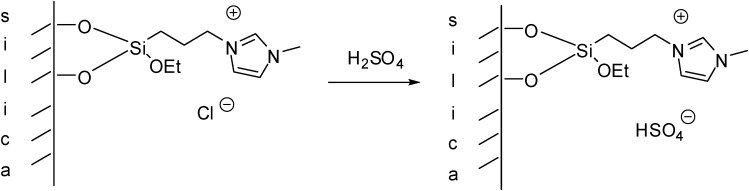
Preparation of a SILP with a Brønsted acidic IL grafted on a silica support.

The simplest method is the synthesis of an IL with a (trialkoxysilyl)-alkyl side chain that can be grafted on the support (as depicted in Scheme 2). Brønsted acidity is due to the [HSO_4_]^−^ anion that can be introduced by ion exchange (Scheme 10) [45,46,47,48,49,50]. In these cases, a mesoporous silica of ordered structure was used as the support, obtained by the condensation of tetraethoxysilane (TEOS) in the presence of the structure directing agent cetyl-tributylammonium bromide.

The attachment of the IL on the silica surface was confirmed by ^29^Si CP MAS NMR. The appearance of a signal at −65 ppm proved the silanisation of silica, but the relatively large signal at −110 ppm suggested there was still a large amount of unreacted hydroxyl groups on the surface. Despite the significant line broadening in the solid-state ^13^C CP MAS NMR spectrum, carbons of the IL could be identified. The lack of signals of the OEt group indicated that the IL was attached to the surface. SEM analysis showed that the structure of the support was not destroyed after the immobilisation of the IL [45].

Magnetic Fe_3_O_4_ nanoparticles with surface OH groups [51] were functionalised by the same method. Immobilisation was proved by CHN analysis and FT-IR. In the IR spectrum, appearance of the peaks at 1036 cm^−1^ (Si–O stretching), 2915 and 2848 cm^−1^ (–CH_2_ stretching) and at 1214 and 1126 cm^−1^ (S=O stretching) confirmed the attachment of the IL. The SEM image showed that the catalyst was made up of uniform nanometer sized particles around 70 nm in diameter, a higher value than that measured for the original magnetic nanoparticles (20 nm). XRD indicated the retention of the crystalline cubic spinel structure. Saturation magnetisation value was found to be lower than that of the bare magnetic nanoparticles due to the silica coating and the layer of grafted catalyst. 

**Scheme 11:**
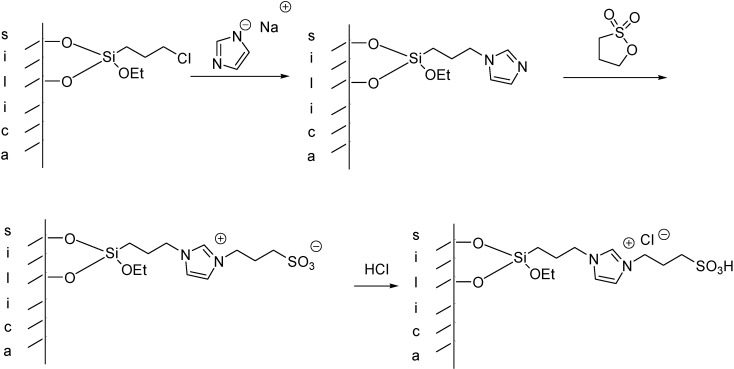
Synthesis of a silica supported IL with a Brønsted acidic side chain [52].

Imidazolium ILs with a Brønsted acidic side chain (such as ILs with a [C_1_(HSO_3_)^4^C_4_im]^+^ cation) are generally prepared by the reaction of the imidazole derivative with 1,4-butanesultone, or 1,3-propanesultone. Immobilisation of these ILs can be carried out in a stepwise manner, *i.e.*, by the treatment of chloropropylated silica with the sodium salt of imidazole [52] or benzimidazole [53,54], followed by the reaction with an alkanesultone that leads to a zwiterrionic structure. Subsequent treatment with HCl results in the formation of a supported Brønsted acidic IL (Scheme 11).

The FT-IR spectra of the SILP material showed the typical peaks of imidazole/benzimidazole moieties and alkyl chains. Two important peaks at 1088 cm^−1^ and 1165 cm^−1^ were assigned to S=O stretching vibration proving the incorporation of the sulfonic acid moiety [54].

**Scheme 12:**
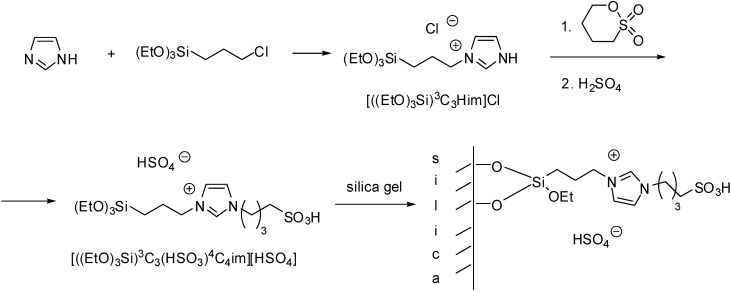
SILP material prepared by grafting of a dual acidic IL on a silica support [55].

In case of dual acidic ILs, with acidic HSO_4_^−^ anions beside the alkanesulfonic acid chains, the supported material was obtained by the grafting of the ready-made IL on silica gel (Scheme 12) [55], a nano-silica material [56] or on silica-coated magnetic nanoparticles [57,58]. The particle size of the silica gel supported IL was found to be similar to that of silica gel but the surface morphology of the two samples was different. Instead of the smooth surface of SiO_2_, the SEM image of SILP showed small aggregates on the surface of silica gel supported IL [55]. Similarly, the surface of the functionalised nano-silica was aggregated but it retained its nano nature [56]. TEM image of the magnetic nanoparticles showed a dark nano-Fe_3_O_4_ core surrounded by a grey silica shell about 3–5 nm thick. The average size of the obtained particles was 20–30 nm [57].

**Scheme 13:**
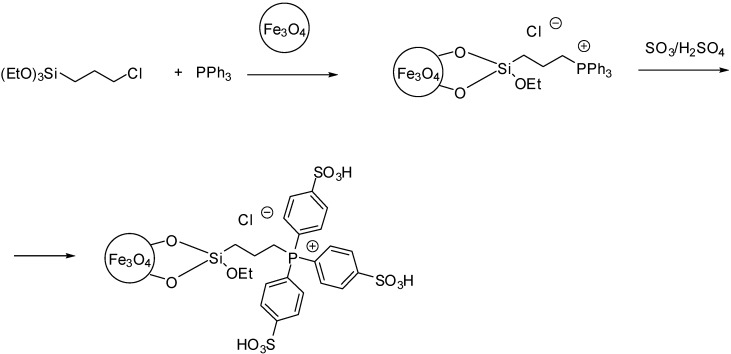
Preparation of phosphonium IL functionalised magnetic nanoparticles [59].

Magnetic nanoparticles were also coated with a phosphonium IL. The acidic side chain was introduced by sulfonation of the phenyl groups of the triphenylphosphonium ion (Scheme 13) [59].

The advantages of magnetic nanoparticles was maintained after the grafting, the particle diameters of the nanoparticles remained in the range of 8–13 nm. As it could be expected, a slight decrease of the saturation magnetisation of the nanoparticles was observed that was due to the successful grafting of ionic liquids on the surface [59].

Beside chloropropylated materials, mercaptopropyl silica was also proved to be a useful material in the synthesis of acidic SILPs. The covalent bond was formed by a radical chain transfer reaction between the modified silica gel [11,30,31] or a thiol functionalised mesoporous silica material [60] and an IL with an unsaturated side chain, such as [(C_1_=C_2_)(HSO_3_)^4^C_4_im][OTf], in the same procedure used for the immobilisation of Lewis acidic sulfonyl chloride derivatives (see Scheme 6). Interestingly, the IL loading of the SILPs obtained with the Brønsted acidic ILs [(C_1_=C_2_)(HSO_3_)^3^C_3_im][OTf] or [(C_1_=C_2_)(HSO_3_)^4^C_4_im][OTf] was more than two times higher (0.56, 0.58 mmol/g, respectively) than their Lewis acidic sulfonyl chloride counterparts [(C_1_=C_2_)(ClO_2_S)^3^C_3_im][OTf] and [(C_1_=C_2_)(ClO_2_S)^4^C_4_im][OTf] (0.25 and 0.18 mmol/g, respectively) [30].

A similar procedure was used for immobilisation of [(HSO_3_)^3^C_3_(C_1_=C_1_)im][HSO_4_] in a mesoporous thiol-functionalised silica [60] and [(C_1_=C_2_)(HSO_3_)^4^C_4_im][OTf] on a magnetic mesoporous material with cobalt ferrite nanoparticles embedded in silica [61]. In the latter case, a partial substitution of the hydrogen ions of the sulfonic acid group by metal ions was observed during the procedure, so the supported IL was dispersed into a dilute H_2_SO_4_ solution of dichloromethane and ethanol to re-establish the SO_3_H functionalities. As another consequence, the exchange between the hydrogen ions and metal ions led to a corrosion of CoFe_2_O_4_ and might have contributed to the decrease of saturation magnetisation of the SILP material. The IL loading of the SILP was found to decrease with increasing number of SH groups. This phenomenon was explained by a decrease in the average pore diameter of the mesoporous material with increasing SH loading. This led to less facile diffusion of the IL to the pores of the support due to the high mass-transfer resistance induced by the small pore diameters.

A triethoxysilyl substituted Brønsted acidic IL ([((EtO)_3_Si)^3^C_3_(HSO_3_)^3^C_3_im][HSO_4_]) was used as the organic component for the preparation of a mesoporous silica material by the sol-gel method [62].

#### 2.2.2. Immobilisation of Brønsted Acidic ILs on Organic Supports

**Scheme 14:**
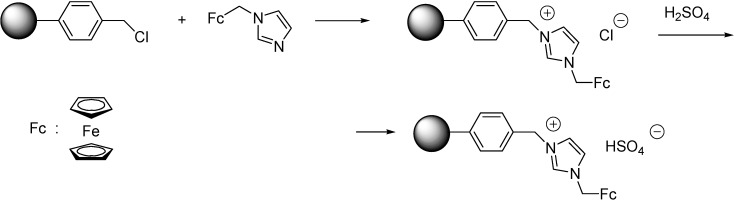
Grafting of a ferrocenyl IL on Merrifield resin [63].

When using polymeric supports, immobilisation can be carried out easily on a Merrifield resin. The ferrocenyl IL [FcC_1_Him][Cl] (Scheme 14) was obtained by the quaternisation of the imidazole derivative by the chloromethyl groups of the polymer [63]. The HSO_4_^−^ ion was introduced by anion metathesis reaction with concentrated sulfuric acid. The procedure was followed by Raman spectroscopy. The intensities of bands at 639 cm^−1^ (C-Cl stretching band) and 1266 cm^−1^ (wagging bands of CH_2_-Cl) were found to diminish while the peaks at 451 cm^−1^ (Fe-Cp stretching band), 1141 cm^−1^, 1451 cm^−1^, 1535 cm^−1^ (ring stretching modes of imidazolium ring), 3138 cm^−1^ and 3191 cm^−1^ (C-H stretching of Cp rings) increased in intensity showing the success of the grafting. The product of the anion metathesis reaction showed diagnostic bands of 910 cm^−1^ (SOH stretch), 1031 cm^−1^ (SO_2_ symmetrical stretch) and 1193 cm^−1^ (SO_2_ asymmetrical stretch). SEM images indicated that the covalent attachment of IL had no influence on the morphology of the support. TGA measurements showed that the SILP material began to decompose at slightly lower temperature than the Merrifield resin, but still could endure about 210 °C temperature with only a small loss of weight.

**Scheme 15:**
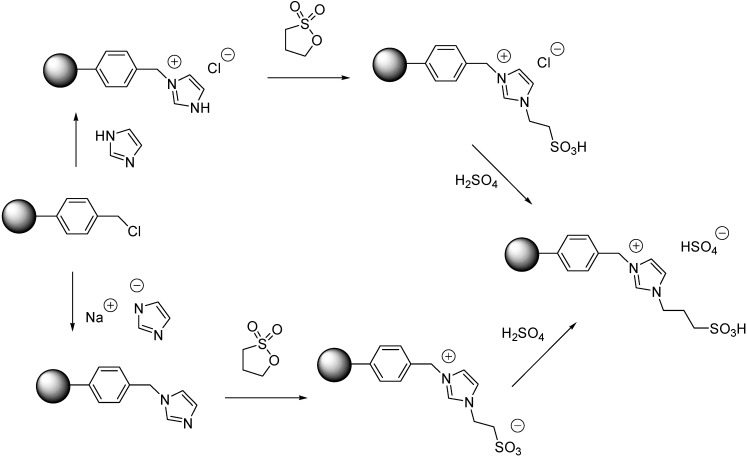
Procedures for the immobilisation of dual acidic ILs on polymers [64,65].

Supported ILs with sulfonic acid side chains were obtained either by quaternerisation of imidazole [64], or by a nucleophilic substitution reaction with the sodium salt of imidazole (Scheme 15) [65]. The next step, the reaction with 1,3-propanesultone resulted in the formation of a supported IL and in a zwitterionic derivative, respectively. Finally, a reaction with sulphuric acid led to the acidic SILPs in both cases.

In contrast to the supported ferrocenyl IL, based on the TG-DSC analysis it was concluded that the immobilised dual acidic IL improved the thermal stability of the original polymer [64].

A hybrid organic/inorganic support, that may offer the physical and chemical stability of pure silica gel and can be modified as a polymer, was also used in the preparation of a SILP catalyst [66]. The support was obtained by the polymerisation of 3-mercaptopropyltrimethoxysilane modified vinyl benzyl chloride, followed by co-condensation with tetraethoxysilane. The pre-formed IL was grafted on the polymer that coated the silica particles via alkylation of the imidazole ring of the IL by the benzyl chloride moieties of the support. The SEM image of the product was identical with that of a compact polymer. Unfortunately, no thermal analysis data were reported to support the enhanced stability of the hybrid material.

**Scheme 16:**
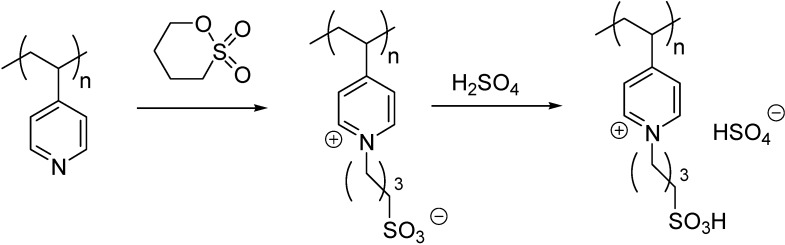
Preparation of SILP starting from poly(4-vinylpyridine) [67,68].

Supported dual acidic ILs were also obtained from polymers incorporating heterocyclic moieties, such as poly(4-vinylpyridine) [67,68] or a melamine resin [69]. The reaction of the heterocycles with 1,4-butanesultone led to a zwitterionic structure that was converted to the IL with sulphuric acid (Scheme 16). The SILP materials were found to be stable below 200 °C [68] and 300 °C [69], respectively.

In the SEM photograph of the polyvinylpyridine-supported IL, a uniform surface appeared that showed that the pores of the polymer were filled with the IL moiety [68].

**Scheme 17:**
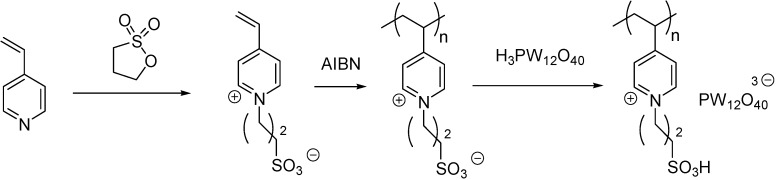
Preparation of a SILP with heteropolyanions by polymerisation.

A polymeric SILP was obtained by the polymerisation of the zwitterions obtained from 4-vinylpyridine and 1,3-propanesultone (Scheme 17) [70]. The acidic moiety was formed by the reaction of a heteropolyacid. Comparing the FT-IR spectra of the original heteropolyacid and the SILP, a slight shift of the Keggin bands was observed showing a strong ionic interaction between the polymeric cations and heteropolyanions. The decomposition temperature of the polymer was around 300 °C which is considerably lower than that of the heteropolyacid.

During the copolymerisation of styrene and acidic 1-vinylimidazolium ILs ([(HSO_3_)^4^C_4_(C_1_=C_1_)im][OTf]) [71,72], the use of a proper ratio of the monomers was found to be essential. An initial ratio of the IL below 5% resulted in the formation of a white powder, but the product became viscous when the initial ionic liquid/styrene ratio was too high [71].

[((EtO)_3_Si)^3^C_3_C_1_im]Cl was grafted with the help of the (trialkoxysilyl)-alkyl side chain on cellulose, activated by a NaOH solution [73]. The attachment of the IL on the surface was proved by the appearance of the characteristic stretching vibration bands due to the imidazole ring at 1644 and 1564 cm^−1^ (C=N and C=C vibration peaks).

### 2.3. Immobilisation of Dual Acidic ILs with Brønsted Acidic and Lewis Acidic Sites

As both Lewis and Brønsted acids can be important for acid-catalysed reactions, the synthesis of SILP catalysts that combine the advantages of Lewis acidic and Brønsted acidic sites can raise interest. Immobilisation of such ILs usually followed the procedures discussed in the previous sections (Table 3).

The Brønsted acidic IL [((EtO)_3_Si)^3^C_3_(HSO_3_)^3^C_3_im][HSO_4_] was grafted on a Fe-incorporated SBA-15, prepared by a sol-gel method from tetraethoxysilane in the presence of iron(III) nitrate [74]. Comparison of FT-IR spectra obtained before and after adsorption of pyridine made it possible to identify the nature of the acid sites on both of the support and on SILP. A new peak in the spectrum of the support after pyridine adsorption at 1449 cm^−1^ was assumed to suggest a Lewis acid site. The appearance of an additional absorption band at 1544 cm^−1^ in the SILP material, owing to a Brønsted acidic site, supported its dual acidity. XRD patterns indicated a long-range ordered structure and well-formed hexagonal lattice of the mesoporous material.

**Figure 2:**
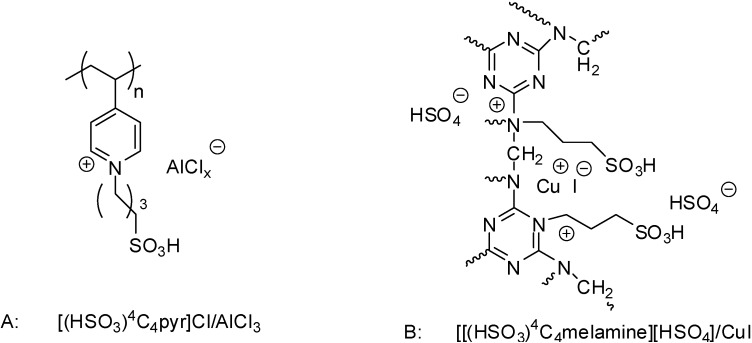
Supported ILs with both Lewis acidic and Brønsted acidic sites.

Poly(4-vinylpyridine) could be converted to a dual acidic IL (Figure 2A) by a similar route as depicted in Scheme 16 but by the use of HCl instead of H_2_SO_4_ [75]. Lewis acidity was achieved by the addition of a proper amount of AlCl_3_. The catalyst was found to be reasonably stable to air and moisture and could be kept for more than a year without appreciable change in its efficiency.

Another dual acidic IL was obtained form a melamin resin modified with 1,4-butanesultone and CuI (Figure 2B) [76]. Immobilisation of copper was proved by XRD. Regular particles of size of 2.5–4.7 μm were obtained after the addition of PEG-2000 that could stabilise the first formed small particles and prevented them from further growth.

**Table 3:** Preparation of dual acidic SILPs with both Lewis and Brønsted acidity and their application in catalytic reactions.

Ionic Liquid	Supporting Method	Support	Catalytic Reaction	Ref.
[((EtO)_3_Si)^3^C_3_(HSO_3_)^3^C_3_im][HSO_4_]	grafting (condensation)	Fe-incorporated SBA-15	esterification	[74]
[(HSO_3_)^4^C_4_pyr]Cl/AlCl_3_	grafting (alkylation	poly(4-vinylpyridine)	domino Knoevenagel-type condensation/Michael reaction	[75]
[[(HSO_3_)^4^C_4_melamine][HSO_4_]/CuI	grafting (alkylation)	melamine-formaldehyde resin	acetal formation	[76]

## 3. Catalytic Reactions in the Presence of Acidic SILPs

The acidic SILPs, described in the previous sections, were tested in several acid-catalysed organic reactions, including such relatively simple procedures as Friedel-Crafts reactions, esterification or acetal formation, as well as elaborated multicomponent reactions. As the following examples show, they were proved to exhibit outstanding activity together with reasonable recyclability in most cases. 

**Figure 3:**
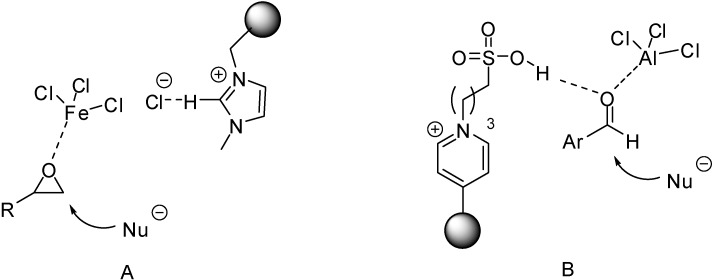
Activation of epoxides (**A**) and aldehydes (**B**) by supported Lewis acidic ILs.

Although mechanistic investigations have not been carried out, most of the authors suggested mechanisms pretty similar to those proposed for acid catalysed reactions under homogeneous conditions. In case of Lewis acidic SILPs, substrates are thought to be activated by the coordination of the Lewis acidic species (Fe(III) [33] or Al(III) [75], Figure 3). The effect may be enhanced by the presence of Brønsted acidic moieties, such as in case of [(HSO_3_)^4^C_4_pyr]Cl/AlCl_3_ [75] (Figure 3B).

**Figure 4:**
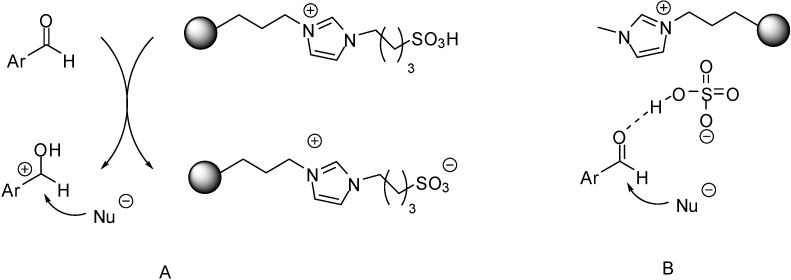
Activation of aldehydes via proton transfer from a SO_3_H-functionalised cation (**A**) [46], and via coordination of a HSO_4_^−^ anion (**B**) [56] of acidic SILPs.

In case of Brønsted acidic SILPs, activation of substrates is thought to proceed either via protonation (Figure 4A) [39,43,51,53,56,58,62,63] or via hydrogen bond formation, without assuming a real proton transfer from the catalyst to the substrate (Figure 4B) [46,47,48,54,55,57,64,67,68] in the presence of [HSO_4_]^−^ anions [46,47,48,51,63], SO_3_H-functionalised cations [43,53,54,56], or both [39,55,57,58,62,64,67,68].

### 3.1. Friedel-Crafts Reactions

The first application of a chloroaluminate IL supported on silica in a Friedel-Crafts reaction was reported by Hölderich [21]. The catalysts were prepared by the wet impregnation method and were tested in the alkylation of benzene and toluene with dodecene at 80 °C. A high selectivity towards monoalkylated products was observed in each case. The increase of the AlCl_3_ content of the SILP led to an increase of dodecene conversion and higher selectivity towards the C-alkylated product due to an increase in the acidity of the system. A filtration study of the catalyst showed no further reaction after filtration, but upon recycling the catalysts, a strong deactivation was observed possibly due to residual moisture in the filters. When extreme care was taken to ensure completely dry conditions, the catalysts were active in a second run. An additional factor in deactivation was thought to be the strong adsorption of dodecene on the surface of the catalyst, followed by its oligomerisation that caused blocking of the active sites. This can be the reason for the slow deactivation of the catalyst with time on stream during liquid continuous phase experiments. Gas phase reactions led to only a small dodecene conversion.

Chloroaluminate ILs grafted on MCM-41 through the side chain of the cation (see Scheme 2) was proved to be superior, especially at lower temperatures and with a lower catalyst ratio [18]. This observation was attributed to the higher surface area of the MCM-41, and the resulting higher amount of immobilised IL on this support.

The [((EtO)_3_Si)^3^C_3_C_1_im][FeCl_4_] IL grafted on MCM-41 was used by Yu *et al.* for the alkylation of benzene by benzyl chloride [28]. With an increase in the FeCl_3_ ratio from 1.0 to 1.5, compared to the IL, the conversion of benzyl chloride increased from 2% to 100% in 45 min reaction time. The most important advantage of immobilisation was the dramatically improved recyclability of the SILP compared to the IL itself: conversion changed from 100% to 94% in ten cycles, while a drop from 100% to 19% was observed in the unsupported case in five cycles. Also, reusability of FeCl_3_ supported on MCM-41 was shown to be clearly inferior to the SILP catalyst.

The immobilised chloroindate(III) ionic liquid, prepared by a sol-gel method from [((EtO)_3_Si)^3^C_3_((EtO)_3_Si)^3^C_3_im]Cl and tetraethoxysilane (see Scheme 5) showed an increased resistance to water compared to a similar catalysts obtained with FeCl_3_ [29]. When benzene, saturated with water at room temperature, was used as the substrate in the benzylation reaction, the former catalyst showed no loss of activity in contrast to the latter. Interestingly, the SILP catalyst with chloroindate anions exhibited higher activity even under anhydrous conditions, although FeCl_3_ possesses stronger acidity than InCl_3_. Besides, it could be recycled efficiently, the conversion was found to decrease only slightly from 100% to 97% and the selectivity to the monoalkyl product kept unchanged.

**Scheme 18:**
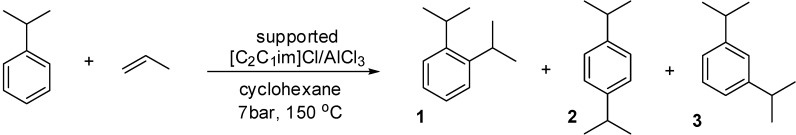
Friedel-Crafts alkylation of cumene.

Wasserscheid *et al.* developed a new method for the preparation of supported chloroaluminate catalysts by the pretreatment of the support with the same IL before immobilisation of the IL catalyst itself [26]. The catalyst showed higher activity in isopropylation of cumene (Scheme 18) than the liquid-liquid biphasic system of the unsupported IL. At the same time, a different selectivity of the SILP catalyst was also observed, leading to the preferential formation of the kinetically favoured products, *para*-diisopropylbenzene (**2**) and *ortho*-diisopropylbenzene (**1**). In contrast, in the liquid-liquid biphasic system the thermodynamically most stable *meta*-diisopropylbenzene (**3**) was the main product. In the recycling experiments of the SILP catalyst, 84% of cumene was still possible to be converted to the products in only 3.5 min reaction time even in the third reuse. Selectivity to *meta*-diisopropylbenzene decreased from run to run that was attributed to a change in the nature of acidic species due to an increasing contamination of the SILP catalyst with moisture from air. 

The same SILP material was proved to be a very active and selective catalyst in the continuous gas-phase Friedel-Crafts isopropylation of toluene and cumene [77]. The catalyst was found to be superior to those obtained with other supports (calcined silica gel or silanised silica gel).

Detailed kinetic studies and analysis of deactivation behaviour along the reactor length, in a catalytic reactor with four independent catalyst beds, showed that beside the water impurity of feedstock, the aromatic/alkene ratio is the other relevant factor influencing the stability of the catalyst. A moderate IL loading with considerably high aromatic/alkene ratio were found to be the best conditions to suppress consecutive alkylation reactions that filled the catalyst pores with products of low volatility. With technical grade reactants and low toluene/propene ratio the catalyst system lost 30% of its initial activity within the first 60 h time-on-stream. At the same time, with a dry feedstock of high toluene/propene ratio, the catalyst was found to be stable over at least 210 h time-on-stream with high selectivity to the mono-alkylated product (>95%) and excellent selectivity to meta-cymene within the cymenes (up to 80%). 

The activity of a SILP obtained by the impregnation of silica with the Lewis acidic IL [C_4_C_1_im]Cl/FeCl_3_ was tested in Friedel-Crafts acylation of various aromatic compounds (benzene, toluene, mesitylene, anisole, *m*-xylene) with acetil chloride or anhydride [23]. Reactions in batch and continuous liquid phase resulted in lower conversions but higher selectivities than homogeneously catalysed reactions. In gas phase reactions a deactivation through heavier products took place. ICP measurements showed a strong leaching of the IL probably due to a complex formation between the metal halide and the carboxylic groups of the reactants. 

### 3.2. Nitration

Brønsted acidic [(C_1_=C_2_)(HSO_3_)^4^C_4_im][OTf] and [(C_1_=C_2_)(HSO_3_)^3^C_3_im][OTf] as well as Lewis acidic [(C_1_=C_2_)(ClO_2_S)^4^C_4_im][OTf] and [(C_1_=C_2_)(ClO_2_S)^3^C_3_im][OTf], grafted on 3-mercaptopropylated silica by a radical chain reaction (see Scheme 6), were found to be effective catalysts for nitration of benzene with 62% nitric acid and to exhibit higher activity than their IL precursor [30]. The nitration product was separated by decantation and the supported ionic liquid catalysts could be reused. Only a little loss of activity, with a drop of the conversion from 62% to 58% after three cycles, was observed.

**Scheme 19:**
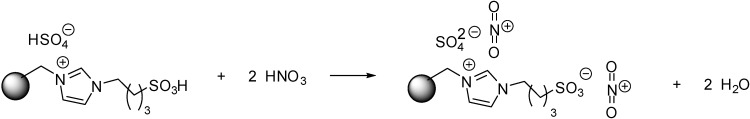
Formation of nitronium ions from HNO_3_ in the presence of supported [(HSO_3_)^3^C_3_Him][HSO_4_].

Nitration of toluene with 95% nitric acid was attempted using a Brønsted acidic IL, [(HSO_3_)^3^C_3_Him][HSO_4_], supported on chloromethylated polystyrene, as catalyst [65]. The nitronium ion was thought to be generated by the reaction of HNO_3_ with activated H^+^ in the SILP catalyst (Scheme 19).

Although the yield of mononitrotoluene was 99%, some visible particles were peeled off from the SILP, probably due to the concentrated nitric acid. In the presence of 68% nitric acid, the yield dropped to 75% but the catalyst could be reused with a small loss of activity (67% yield in the fifth run). It should be mentioned that despite the use of a more diluted nitric acid, some degradation of the polymer could still be observed. The catalyst exhibited higher activity and better *para*-selectivity than its homogenous counterpart. The *para*-selectivity was explained by the assumption that the nitronium ion, associated with the immobilised IL, exerted a greater steric hindrance than that connected to the unsupported IL.

### 3.3. Alkylation and Oligomerisation

A method for alkylation of isobutane with 2-butene in the presence of SILP catalysts was patented in 1993 by IFP [20]. The catalysts, obtained by the impregnation of silica with imidazolium or pyridinium chloroaluminate ILs, showed good activity and C8 selectivity.

A SILP catalyst prepared by grafting the [((EtO)_3_Si)^3^C_3_C_1_im]^+^ cation on silica was applied in the alkylation of a reaction mixture of pure isobutane and an industrially obtained raffinate II [27]. The catalyst obtained with grafting was found to be superior to other solid acid catalysts tested, such as SAC 13 or zeolite H-Beta. The catalyst could be reused with some loss of activity and aluminum leaching to the liquid phase was found to be negligible. However, the amount of liquid products obtained was low, due to the low olefin-content of the feed.

Supported chloroaluminate ILs were tested in the trimerisation of isobutene in a feed containing a C_4_ mixture [22]. Both the support and immobilisation methodology were found to play a crucial role in the reaction. Exclusive isobutane/butene alkylation took place in the presence of ILs immobilised by impregnation on glass or molecular sieves, as well as with a SILP obtained by grafting the cation on the silica support. In contrast, catalysts obtained by impregnation of silica supports with [C_4_C_1_im]Cl showed excellent oligomerisation activity. Total conversion of isobutene and 91.4% selectivity of trimers were obtained at 50 °C.

The results can be interpreted based on the different structure of supported ILs as described in Section 2.1.(see Scheme 1 and Scheme 2). The covalent Si-O-Al bonds, formed during the impregnation method, were assumed to adsorb butenes instead of isobutane on the surface of the catalyst and promote oligomerisation. In case of the grafted IL, no such bonds are formed because Si-OH groups takes part in a condensation reaction with the Si(OEt)_3_ moieties of the cation.

From the careful investigations of SILP structures carried out by Hölderich *et al.* [18] it is known that the presence of Al_2_Cl_7_^−^ ions can be observed in grafted chloroaluminate ILs, similarly to the unsupported ILs. In contrast, they are totally absent from SILPs obtained by impregnation of silica, due to the formation of Si-O-Al bonds. Although no structural investigations of the SILPs obtained on supports such as glass or activated carbon have been carried out, the existence of Al_2_Cl_7_^−^ ions can be presumed as no support-O-Al bonds can be formed here. Then, from the catalytic results it can be concluded that the presence of Al_2_Cl_7_^−^ ions with high Lewis acidity leads to an oligomerisation reaction, while in their absence an alkylation takes place. 

Silica-supported SO_3_H-functionalized IL catalysts were used in the oligomerisation of isobutene [41]. The catalysts were obtained by impregnation of silica gel with Brønsted acidic ILs with [C_1_(HSO_3_)^4^C_4_im]^+^ or [C_4_(HSO_3_)^4^C_4_im]^+^ cations. It was shown that various factors, such as the properties and pretreatment of the solid support, the choice of cation and anion, as well as the reaction time and temperature affected the outcome of the reaction considerably. Oligomerisation of isobutene, carried out at 60 °C, led to the formation of C_8_ products with very good selectivity in each case. An increase in the temperature and/or reaction time led to an increase in the ratios of higher oligomers with selectivities for C_12_+C_16_ products up to 85%. The catalysts comprising a trifluoromethanesulfonate anion could be reused several times without loss of activity. Total catalyst leaching of eight successive runs was 2.0% of the original load. At the same time, a quick inactivation of the catalysts obtained from ILs with hydrogensulfate anions was observed. The TON and TOF values of the SILP catalysts were found to be ten times higher than those of the same ILs. 

A close relationship between catalytic activity and catalyst morphology was observed [42]. Silica with mesoporous structure was able to adsorb a higher amount of the IL and produced SILP materials with higher catalytic activity at identical catalyst loadings. The formation of a stable film was a prerequisite for an unvarying selectivity of the catalyst, so impregnation at a high temperature was necessary to obtain a suitable composition, especially in case of microporous support materials.

The diffusion of isobutene to the micropores was possibly blocked by the IL that filled the pore, so the contact surface between the IL phase and the organic phase was lower than that of SILP catalysts with mesopores. Also, acid capacity of microporous material was found to be lower, in accordance with the different amounts of adsorbed ionic liquid. Lower contact surface and lower acidity resulted in lower catalytic activity. At the same time, using a proper pretreatment, a stable catalyst with excellent C_12_ selectivity, exceeding even that of the mesoporous material, could be obtained with ILs supported on microporous silica.

### 3.4. Esterification and Transesterification

Both immobilised [(C_1_=C_2_)(HSO_3_)^4^C_4_im][OTf] and [(C_1_=C_2_)(HSO_3_)^3^C_3_im][OTf], as well as their Lewis acidic sulfonyl chloride derivatives ([(C_1_=C_2_)(ClO_2_S)^4^C_4_im][OTf] and [(C_1_=C_2_)(ClO_2_S)^3^C_3_im][OTf]), supported on mercaptopropylated silica, were shown to catalyse the esterification reaction of ethanol with acetic acid with 72%–82% yields [30]. The immobilised ILs exhibited the same catalytic activity as their homogenous counterpart. SILPs with the Lewis acidic ILs were found to be more effective. The supported IL catalysts were separated by simple decantation and could be reused several times without a significant loss of catalytic performance, with a conversion dropping from 82% to 81% in the third cycle. Esterification of several other substrates, including long chain carboxylic acids and alcohols, were also carried out effectively but with longer reaction time and at higher temperature.

Similar activity of pure and polystyrene-grafted [(HSO_3_)^3^C_3_Him][HSO_4_] was observed in the esterification reaction of n-butyl alcohol and acetic acid [64]. Only a 7.3% decrease in the catalytic activity was observed after 13 cycles in case of the supported catalyst. FT-IR and SEM characterisations indicated a change in the size and surface of the reused catalyst. At the same time, supported by FT-IR analysis, the change in catalytic activity was mainly attributed to some *n*-butyl acetate adhering on the surface of reused catalyst, which might cover the active site. The catalyst exerted good catalytic activity for a series of esterification reactions of different alcohols and acids.

Almost the same results were obtained with a SILP catalyst prepared by a radical chain transfer reaction between [(HSO_3_)^3^C_3_(C_1_=C_1_)im][HSO_4_] and mercaptopropylated silica [60]. The only exception is the greater loss of activity of the catalyst compared to the polystyrene-supported derivative [64], showing approximately the same loss of activity in only 6–7 cycles. The decrease in catalytic activity was ascribed to the loss of the IL.

[(HSO_3_)^3^C_3_Him][HSO_4_] grafted on a polystyrene-silica hybrid could be reused in 9 cycles with yields between 90%–92% in the esterification of propanoic acid and methyl alcohol [66]. Excellent yields (88%–94%) were achieved with other alcohols as well as in the esterification of acetic acid. The SILP catalyst showed somewhat better activity than the unsupported IL. 

In contrast to the previous results, the activity of supported catalysts, obtained by the same radical addition reaction with a magnetic mesoporous silica support, was noticeably lower than the corresponding free IL ([(C_1_=C_2_)(HSO_3_)^4^C_4_im][OTf]) in esterification of long chain acids, such as oleic acid [61]. Also, a raise in the carbon number of the straight-chain alcohol reactant caused a significant decrease in the conversion of oleic acid. These two observations demonstrate well the fact that the mass-transfer resistance strongly affected the catalytic activity. Besides, to achieve sufficiently high conversion, the catalyst had to be treated with a dilute H_2_SO_4_ solution to compensate the substitution of hydrogen ions of [(C_1_=C_2_)(HSO_3_)^4^C_4_im][OTf] with metal ions during the preparation of the catalyst. The nitrogen content of the catalyst decreased after the reaction, so no recycling experiments were carried out.

Better results were achieved with a catalyst, bearing both Lewis acidic and Brønsted acidic sites, obtained by grafting [((EtO)_3_Si)^3^C_3_(HSO_3_)^3^C_3_im][HSO_4_] on Fe-incorporated SBA-15 [74]. The cooperation of Lewis acidic and Brønsted acidic sites was demonstrated by the fact that the catalyst gave better results (88% conversion) than IL grafted on SBA-15 (81% conversion), in spite of the higher acidity of the latter. Good results were obtained also with C_3_-C_4_ alcohols as reactants. As another advantage, this catalyst could be reused, although the conversion of oleic acid decreased from 87.7% to 80.8% in six cycles, due to a possible loss of catalyst during the recycling.

**Scheme 20:**
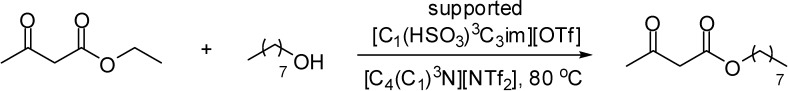
Supported IL-catalysed transesterification of ethylacetoacetate and octanol in an IL solvent.

Excellent results were obtained in the transesterification of ethylacetoacetate and octanol (Scheme 20) in the presence of a catalyst prepared by ion exchange of [C_1_(HSO_3_)^3^C_3_im][OTf] into clay interlayers [43]. Although showing similarly excellent catalytic activities, with TONs of 16,000–19,000 and TOFs of 5333–6333 h^−1^, a higher efficiency of recycling was observed with the SILP catalyst than the unsupported IL. On comparing the results obtained under solventless conditions and in the presence of the same IL as solvent, it was concluded that the superiority of the latter conditions might be due to an assistance of the IL in the transport of reactants into the layers of clay.

A benzimidazolium IL ([((EtO)_3_Si)^3^C_3_(HSO_3_)^3^C_3_bim]Cl) grafted on silica was found to be a highly efficient catalyst for the synthesis of biodiesel from non-edible oils [53]. Castor, jatropha, and neem oil with a high free fatty acid content were reacted with methanol. For the conversion of jatropha and neem oil a longer reaction time was required because of the slow mixing of the oils with methanol. The reaction of the more soluble castor oil was more facile. Under optimal reaction conditions approximately 95% yield was achieved under mild conditions and the catalyst was recycled and the products were formed with 83% yield even in the 5th run.

### 3.5. Acetal Formation

**Scheme 21:**
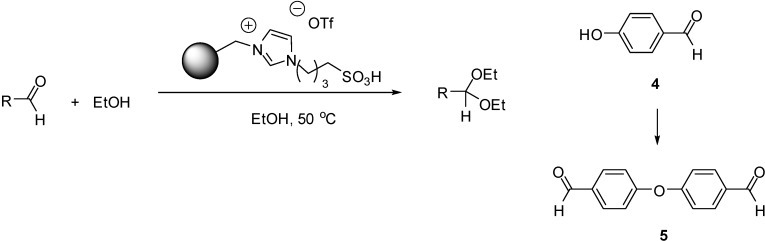
Acetal formation of aldehydes and side reaction of p-hydroxybenzaldehyde (**4**).

Catalysts obtained by copolymerisation of styrene and [(HSO_3_)^4^C_4_(C_1_=C_1_)im][OTf] (with IL ratios of 0.45 and 0.81 wt %) were very effective catalysts for the conversion of a great variety of aldehydes to the corresponding acetals with ethanol as the reaction partner (Scheme 21) [71]. High yields of acetals were obtained both from aliphatic and aromatic aldehydes in 0.5 h at 50 °C. The only exception was the reaction of *p*-hydroxybenzaldehyde (**4**) that led to the formation of 4,4'-oxydibenzaldehyde (**5**) as the only product instead of the corresponding acetal. In the recycling experiments the SILP with the lower IL content exhibited nearly no loss of activity in the second run (with 95% and 97% conversion in the reaction of *n*-hexanal with ethanol in the first and second run, respectively). At the same time, a remarkable decrease in activity (from 94% to 75% in the second cycle) was observed with the other SILP, probably due to a decomposition of the catalyst.

A Lewis acidic tetrachlorogallate SILP, on a support obtained by the copolymerisation of [C_4_(C_1_=C_1_)im]Cl and styrene, was proved to be even more efficient [36]. Although at a 400:1 substrate/catalyst ratio both the Brønsted acidic and the Lewis acidic SILP gave a high yield of the product (>95% in the reaction of benzaldehyde with methanol). At the same time, a 94% yield was obtained with the latter catalyst at a substrate/catalyst ratio as high as 2000:1, while the former provided only 35% yield. Another tetrachlorogallate IL ([((EtO)_3_Si)^3^C_3_C_1_im]Cl/GaCl_3_) supported on silica was also proved to be less active leading to a 75% yield of the acetal with the lower substrate/catalyst ratio. 

Sulfonation of triphenylphosphonium chloride ([((HO_3_S)C_6_H_4_)_3_((EtO)_3_Si)^3^C_3_P]Cl) grafted on magnetic nanoparticles led to a catalyst that can be separated from the reaction mixture by simple magnetic decantation by using a permanent magnet [59]. The catalyst provided the products in 94%–96% yields in the reactions of cyclohexanone, 2-butanone or propanal with ethyleneglycol, but at higher temperature and in longer reaction time (80 °C 2 h), than the tetrachlorogallate SILPs [36]. At the same time it permitted an efficient recycling with a yield above 90% even in the 5th cycle. The X-ray diffraction patterns and average particle diameters of the samples revealed no obvious changes after the fifth run.

Acetals were obtained in 85%–98% yields in 0.5–3 h reaction time from benzaldehyde, furfural and phenylacetaldehyde with diols, in the presence of a catalyst obtained by the sol-gel method from [((EtO)_3_Si)^3^C_3_(HSO_3_)^3^C_3_im][HSO_4_] and tetraethoxysilane [62]. Acetalisation was carried out in cyclohexane solution at 110 °C. The catalyst could be reused for 10 times without significant loss of catalytic activity, with a yield above 90% even in the 10th cycle.

Aliphatic aldehydes were transformed to the corresponding acetals smoothly with good to excellent yields (69%–99%) in 2–4 h in the presence of a sulfonated melamine-formaldehyde resin [69]. Ketones, such as cyclohexanone or 2-butanone, gave also good results, though the reactivity of the latter was found to be lower due to a steric hindrance. The TOF value of the new catalyst in the reaction of cyclohexanone and 1,2-ethanediol was considerably higher (50.06 s^−1^) than that of usual catalysts, such as H_2_SO_4_ (8.09 s^−1^) for p-TsOH(28.38 s^−1^). The catalyst exhibited excellent stability with conversions and yields remaining unchanged even in the seventh run.

By the use of a similar resin modified with CuI, conversion of substrates in the ethylene glycol benzaldehyde reaction increased from 86% to 98% with the addition of CuI [76]. The activity was unchanged even after 6 runs, but a 27% leaching of copper was observed after 6 cycles, which obviously leads to a contamination of the product. 

### 3.6. Cleavage or Cycloaddition Reactions of epoxides

**Scheme 22:**
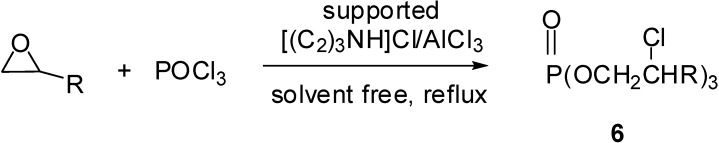
Ring-opening of epoxides with POCl_3_.

To solve problems caused by moisture sensitivity, [(C_2_)_3_NH]Cl/AlCl_3_ was supported on molecular sieves by impregnation and was used efficiently in the ring-opening of various epoxides with POCl_3_ (Scheme 22) [24]. The corresponding tris(chloroalkyl)phosphates (**6**) were obtained in excellent yields and the catalyst could be recycled five times without any loss in its activity.

Even simple and unsupported ILs are widely used as catalysts in the synthesis of cyclic carbonates via cycloaddition of epoxides and CO_2_ [78]. Zn tetrahalide ILs were found to be very effective catalysts but tended to decompose ethylene carbonate and propylene carbonate during the distillation of the product mixture. The use of [poly(1-vinylimidazolium)][zinc tetrahalide] complexes (see Scheme 9) solved this problem and could be recycled efficiently, with TONs changing from 633 to 598 h^−1^ in the 7th run [37].

Polystyrene-supported [C_1_Him][FeCl_4_] was a little less effective with TOFs around 15 h^−1^, but could be used in the synthesis of cyclic carbonates of diverse structure, as well as for the carboxylation of azridines or propargyl amine with CO_2_ [33]. 

**Scheme 23:**
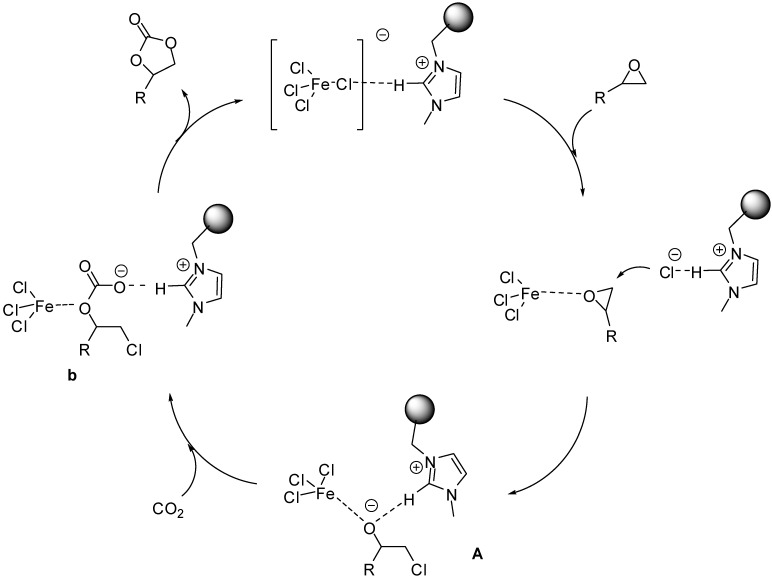
Proposed mechanism for the synthesis of cyclic carbonates in the presence of polystyrene-supported [C_1_Him][FeCl_4_].

According to the proposed mechanism (Scheme 23), the epoxide is activated by the coordination of the Lewis acidic Fe(III) species. Simultaneously, the nucleophilic attack of Cl^−^ on the sterically less hindered β-carbon atom of the epoxide furnishes a ring-opened intermediate (A), which could be stabilised through a H-bonding interaction of the imidazolium ion. Then the insertion of CO_2_ into the intermediate A gives the chloroalkoxy carbonate species B, providing the cyclic carbonate by intramolecular cyclisation.

### 3.7. Condensation Reactions

**Scheme 24:**
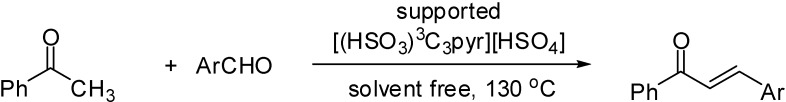
Cross-aldol condensation of acetophenone in the presence of supported [(HSO_3_)^3^C_3_pyr][HSO_4_].

Cross-aldol condensation of aromatic aldehydes and various ketones, such as acetophenone, cyclohexanone and acetone, was carried out with a silica (Aerosil 300) catalyst impregnated with [(HSO_3_)^4^C_4_pyr][HSO_4_] [40]. Under solvent-free conditions, unsaturated ketones could be obtained with complete stereoselectivity from a great variety of aromatic aldehydes in 84%–94% yields after only 30–40 min at 130 °C (Scheme 24). The catalyst was found to be superior upon comparing the results with those reported previously for cross-aldol condensation reactions in the presence of various homogeneous, heterogeneous, and supported catalysts, except for the KF-Al_2_O_3_-catalysed condensation under microwave irradiation. Only a small decrease of activity was observed upon recycling the catalyst, with yields changing from 94% to 89% in five cycles. At the same time, the reactions of cyclohexanone or acetone resulted in a mixture of products due to mono- and double condensation reactions.

Polystyrene supported [C_1_Him]Cl/AlCl_3_ catalysed the Knoevenagel condensation of aromatic and aliphatic aldehydes with ethyl cyanoacetate efficiently [32]. The products were obtained at room temperature in ethanol in good yields (92%–97%, in 0.5–1.4 h). Aliphatic aldehydes were found to be less reactive and the condensation failed to proceed with ketones. The catalyst was recycled with a negligible release of AlCl_3_, with a decrease in the yield from 94% to 90% in the 5th run in the reaction of benzaldehyde. It should be mentioned that no competing Michael addition, observed before with unsupported Lewis acidic IL catalysts, took place here.

**Scheme 25:**
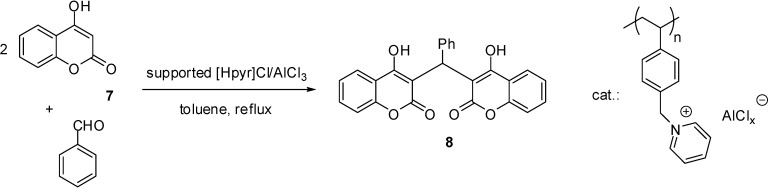
Domino Knoevenagel condensation/Michael addition of 4-hydroxycoumarin (**7**) and benzaldehyde.

Almost exactly the same results were obtained in the presence of a pyridinium functionalised analogue of this SILP [34]. The latter catalyst was also applied in a domino Knoevenagel condensation/Michael addition of 4-hydroxycoumarin (**7**) and benzaldehyde leading to 3,3'-phenylmethylenebis-(4-hydroxycoumarin) (**8**) (Scheme 25). The same domino reaction was extended to other aldehydes including aromatic, aliphatic, heteroaryl and aralkyl derivatives, using a poly(4-vinyl)pyridine supported dual acidic [(HSO_3_)^4^C_4_pyr]Cl/AlCl_3_ catalyst [75]. The presence of AlCl_3_ was shown to be necessary for an efficient reaction. Besides, yield of **8** increased from 67% to 95% when AlCl_3_ mol fraction was increased from 0.4 to 0.7. At the same time, the presence of Brønsted acidic sites really improved catalytic performance leading to the products in 95%–96% yields in reaction times less than an hour at 90 °C. As a comparison, the similar Lewis acidic catalyst [34] produced a 90% yield in refluxing toluene in 1 h.

Reuse of the dual acidic catalyst led to a small drop of activity (from 95% to 90% in the 5th cycle), but almost negligible leaching of aluminum could be observed. Filtration tests proved that that there was no leaching of acid moieties during the reaction, as no activity of the filtrates was observed. The catalyst showed excellent stability by exhibiting almost the same activity after one year storage.

**Scheme 26:**
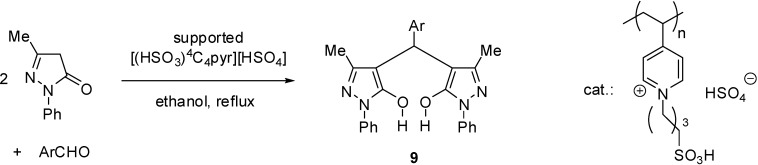
Synthesis of 4,4'-(arylmethylene)bis(3-methyl-1-phenyl-1H-pyrazol-5-ol)s (**9**) by a domino Knoevenagel condensation/Michael addition.

A dual Brønsted acidic IL ([(HSO_3_)^4^C_4_pyr]HSO_4_) supported on poly(4-vinylpyridine) was applied in the synthesis of 4,4'-(arylmethylene)bis(3-methyl-1-phenyl-1H-pyrazol-5-ol)s (**9**, Scheme 26) [67]. Even acid sensitive aldehydes, such as 2-furyl and 2-thienyl carbaldehyde, were smoothly converted into the corresponding products in 90%–97% yields in less than an hour in ethanol reflux. The efficiency of the catalyst was similar to that of other solid acid catalysts in the same reaction, reported before. Starting from benzaldehyde, the yields changed from 95% to 90% in five cycles.

**Scheme 27:**
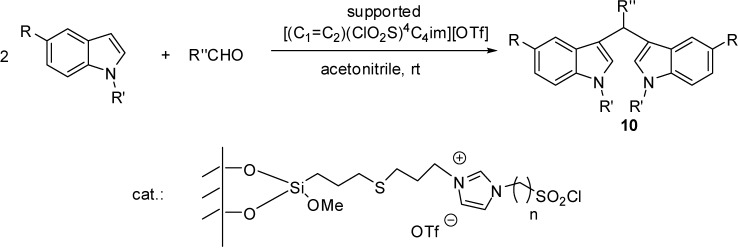
Synthesis of bis(indolyl)methanes (**10**) with a catalyst supported by a radical chain transfer reaction.

Bis(indolyl)methanes (**10**, Scheme 27) could be prepared in the presence of a silica supported Lewis acidic ([(C_1_=C_2_)(ClO_2_S)^4^C_4_im][OTf]) catalyst [31]. It was found to be far more efficient than the analogous supported Brønsted acidic SILP (supported [(C_1_=C_2_)(HSO_3_)^4^C_4_im][OTf]) or the unsupported ILs themselves. The mild conditions, ensured by the catalyst, is well demonstrated by the fact that acetoxy or TBDMS protecting groups in the aldehyde partner remained intact during the reaction. A catalyst obtained by immobilisation of the IL on cylindrical silica pellets was reused four times with an average of 93% yield.

**Scheme 28:**
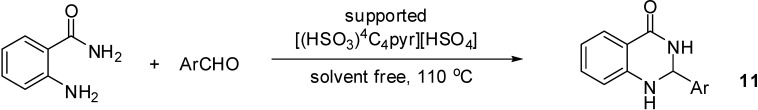
Synthesis of 2,3-dihydroquinazolin-4(1H)-ones (**11**) by a cyclocondensation reaction.

Cyclocondensation of anthranilamides with aldehydes afforded 2,3-dihydroquinazolin-4(1H)-ones (**11**, Scheme 28) with optimum yields of 81%–90% in 10–14 h in the presence of silica (Aerosil 300), impregnated with [(HSO_3_)^4^C_4_pyr][HSO_4_] [39]. Poor recycling results were obtained in polar media, especially water and ethanol, but under solvent-free conditions only a small loss of activity was observed after three successive runs (first use: 90%, third use: 87% yield). The supported catalyst was proved to be superior compared to other solid acid catalysts used generally in similar reactions.

The same condensation was investigated in the presence of a Brønsted acidic IL with [(HSO_3_)^3^C_3_pyr]^+^ cations supported on poly(4-vinylpyridine) [70]. The change of the hydrogen sulfate anion to the anion of a heteropoly acid ([PW_12_O_40_]^3−^) increased conversion from 73% to 87% in ethanol reflux. Under ultrasonic irradiation, yield was further increased to 95% in a considerably reduced time, 10 min instead of 2 h. The catalyst could be recycled six times with negligible loss of activity.

Prins condensation of isobutene with formaldehyde was investigated in the presence of supported Lewis acidic ILs with [SnCl_5_]^−^ anions [25]. The catalyst prepared by the immobilisation of a quaternary ammonium moiety on silica ([((EtO)_3_Si)^3^C_3_(C_3_)_3_N][SnCl_5_]) exerted highest selectivity towards 3-methyl-3-buten-1-ol and could be recycled without a loss of activity. At the same time, in case of silica impregnated with the IL a considerably leaching of tin chloride was observed.

### 3.8. Multicomponent Reactions

**Scheme 29:**
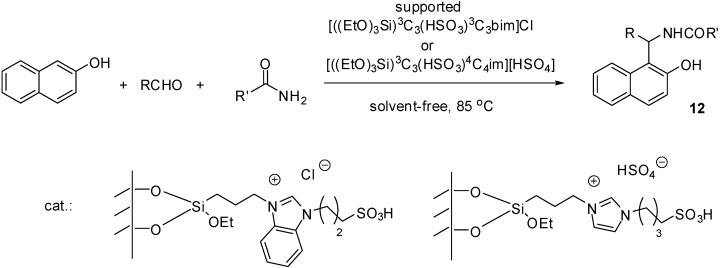
Synthesis of 1-amidoalkyl-2-naphthols (**12**).

1-Amidoalkyl-2-naphthols (**12**) were synthesized by the multicomponent condensation of aldehydes, 2-naphthols and amides (Scheme 29) in the presence of silica supported [((EtO)_3_Si)^3^C_3_(HSO_3_)^3^C_3_bim]Cl [54] and dual acidic [((EtO)_3_Si)^3^C_3_(HSO_3_)^4^C_4_im][HSO_4_] [55]. The latter catalyst showed better performance with a TOF of 6.59 min^−1^ (at 85 °C), in the reaction of 3-nitrobenzaldehdye, acetamide and 2-naphthol, compared to a TOF of 2.84 min^−1^ (at 100 °C) obtained with the former SILP. At the same time, both catalysts afforded the products in excellent yields.

A detailed investigation of catalyst recycling was carried out using the dual acidic SILP. The yield in the above-mentioned model reaction decreased from 94% to 86% in 6 runs. According to FT-IR spectra and SEM images, the recovered catalyst after six runs had no obvious change in structure, morphology and size, in comparison with the fresh catalyst. Hot filtration test proved that no homogeneous species participated in the reaction.

**Scheme 30:**
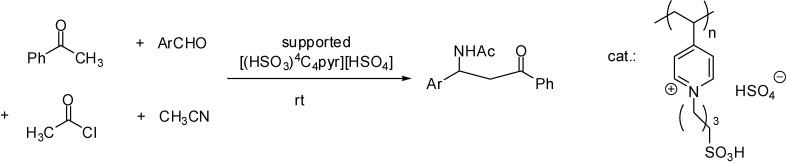
Synthesis of β-acetamido ketones by a multicomponent reaction.

Condensation of aldehydes with acetophenone, acetyl chloride, and acetonitrile led to β-acetamido ketones at room temperature using poly(4-vinylpyridine) supported [(HSO_3_)^4^C_4_pyr][HSO_4_] (Scheme 30) [68]. Aromatic aldehydes afforded the desired products in 90%–94% yields in less than an hour but aliphatic derivatives were found to be unreactive. The catalyst was shown to be more effective than most of other catalysts used before in the same reaction and could be reused five times without appreciable change in its efficiency. The catalyst was also effective for the synthesis of α-aminophosphonates starting from aldehydes, diethylphosphite and aniline.

**Scheme 31:**
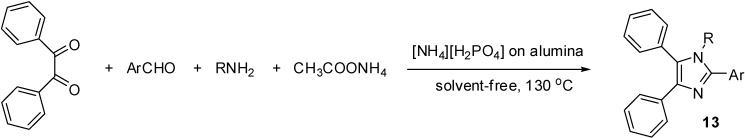
One-pot condensation of aromatic aldehydes, primary amines, and ammonium acetate.

[NH_4_][H_2_PO_4_] supported on alumina was an efficient catalyst in the one-pot condensation of benzil with aromatic aldehydes, primary amines, and ammonium acetate [38]. The 1,2,4,5-tetrasubstituted imidazoles (**13**, Scheme 31) were obtained in 80%–91% yield under optimal conditions. The catalyst could be reused at least three times with a reduction in the catalytic activity from 91% to 86% in the third cycle. Retention of the structure of the catalyst was confirmed by comparing the FT-IR spectra of the recovered and the fresh catalysts.

**Scheme 32:**
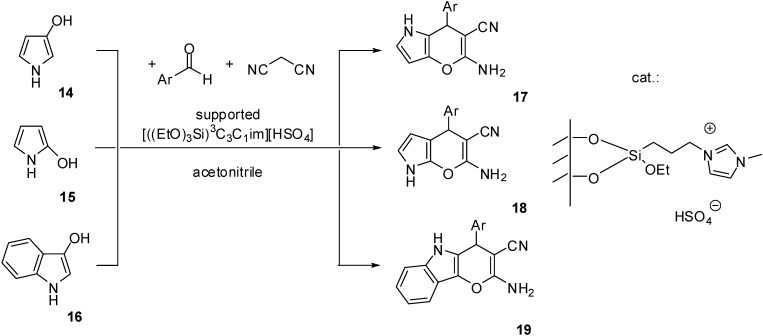
Multicomponent condensation leading to pyrano[3,2-b]pyrrole (**17**), pyrano[2,3-b]pyrrole (**18**) and pyrano[3,2-b]indole (**19**) derivatives.

Pyrano[3,2-b]pyrrole (**17**, Scheme 32) [47], pyrano[2,3-b]pyrrole (**18**) [48] and pyrano[3,2-b]indole (**19**) [46] derivatives were produced by a cyclocondensation of aromatic aldehydes and malononitrile with 3-hydroxypyrrole (**14**), 2-hydroxypyrrole (**15**) or indolin-3-one (**16**), respectively. The silica supported [((EtO)_3_Si)^3^C_3_C_1_im][HSO_4_] catalyst gave very similar results in the three reactions. Immobilisation on silica support slightly enhanced the activity of the corresponding catalyst and shortened the reaction times significantly. The use of polar aprotic solvents, such as acetonitrile, significantly improved the chemical yields and reduced necessary reaction times. The catalyst could be reused with a 6%–9% decrease of its original activity in 4–5 cycles.

**Scheme 33:**
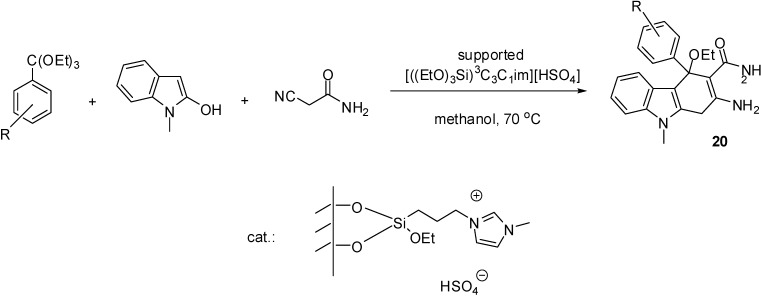
Synthesis of pyrano[2,3-b]indole derivatives (**20**).

Damavandi *et al.* reported on a novel multicomponent synthesis of pyrano[2,3-b]indole derivatives (**20**, Scheme 33), too, with the use of the same catalyst starting from different (triethoxymethyl)arenes, 1-methyl-1H-indol-2-ol and cyanoacetamide [49]. The use of lower temperature (such as methanol reflux) led to the selective formation of the desired products. Both the electronic and steric properties of the starting (triethoxymethyl)arenes greatly influenced the outcome of the reaction, the pyrido-indoles were obtained in 42%–73% yields in 2–11 h.

**Scheme 34:**
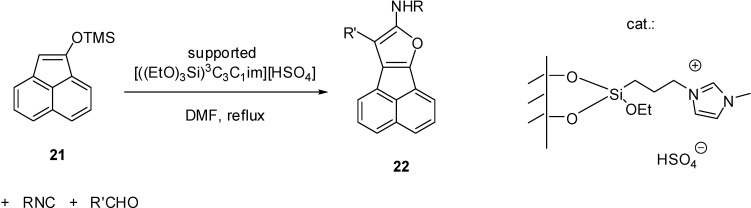
Multicomponent synthesis of acenaphtho[1,2-b]furans (**22**).

Acenaphtho[1,2-b]furans (**22**, Scheme 34) were obtained in a one-pot reaction of (acenaphthylen-1-yloxy)trimethylsilane (**21**), various aldehydes, and isocyanides [50]. The catalytic efficiencies of various ILs, that of the silica support and the grafted [((EtO)_3_Si)^3^C_3_C_1_im][HSO_4_] were compared and the SILP was found to be clearly superior giving the product in 97% yield in a reaction of equimolar amounts of the silyl enol ether of acenaphthylen-1(2H)-one, 2,4-dimethoxybenzaldehyde and cyclohexyl isocyanate.

**Scheme 35:**
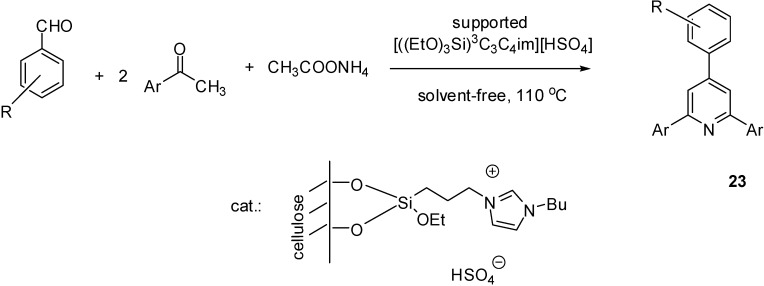
Synthesis of trisubstituted pyridines (**23**) in the presence of [((EtO)_3_Si)^3^C_3_C_4_im][HSO4] grafted on cellulose.

The Brønsted acidic IL [((EtO)_3_Si)^3^C_3_C_4_im][HSO_4_] grafted on cellulose was proved to be a more efficient catalyst for the synthesis of trisubstituted pyridines (**23**, Scheme 35) than other acidic catalysts, such as acetic acid, *p*-toluenesulfonic acid, Amberlyst-15, montmorillonite K-10 or Dowex50WX4 [73]. The products were obtained with 78%–91% yields in 30–40 min with a range of aldehydes and ketones. The decrease of catalytic activity was around 7% of the original value in four cycles.

**Scheme 36:**
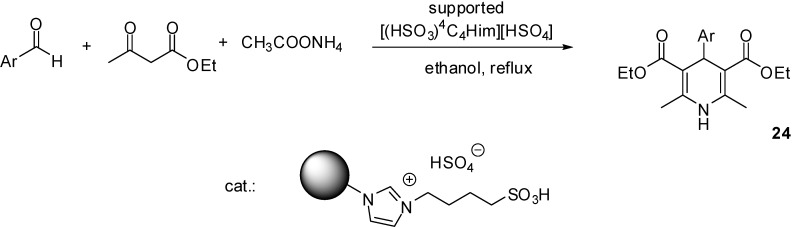
Condensation of aldehydes, ethyl acetoacetate and ammonium acetate to form 1,4-dihydropyridines (**24**).

1,4-Dihydropyridines (**24**, Scheme 36) were obtained with 80%–95% yields by the condensation of aldehydes, ethyl acetoacetate and ammonium acetate in the presence of a SILP obtained by the co-polymerisation of [(HSO_3_)^4^C_4_(C_1_=C_1_)im][HSO_4_] and styrene [72]. The catalyst could be used at least five times with only slight reduction in the catalytic activity in the model reaction of 4-nitrobenzaldehyde with a yield of 95% and 89% in the first and fifth cycles, respectively.

**Scheme 37:**
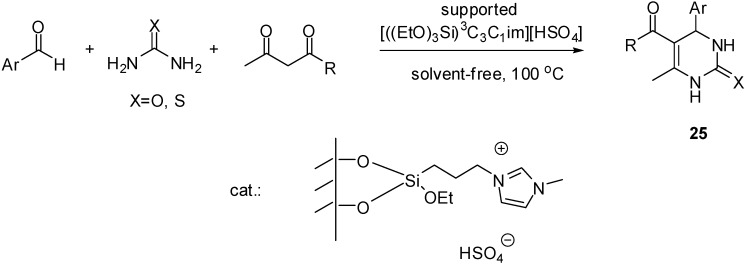
Biginelli synthesis of 3,4-dihydropyrimidin-2(1H)-ones/thiones (**25**).

The Biginelli synthesis of 3,4-dihydropyrimidin-2(1H)-ones or –thiones (**25**, Scheme 37) was performed with [((EtO)_3_Si)^3^C_3_C_1_im][HSO_4_] grafted on magnetic nanoparticles [51]. The reaction led to the products with 90%–98% yields in 20–35 min under solvent-free conditions at 100 °C starting from various aromatic and heteroaromatic aldehydes. The catalyst could be recovered from the reaction mixture by using a simple bar magnet and recycled with an almost negligible decrease in activity (from 95% to 92% in the 8th run) in the model reaction of benzaldehyde, ethyl acetoacetate, and urea. 

**Scheme 38:**
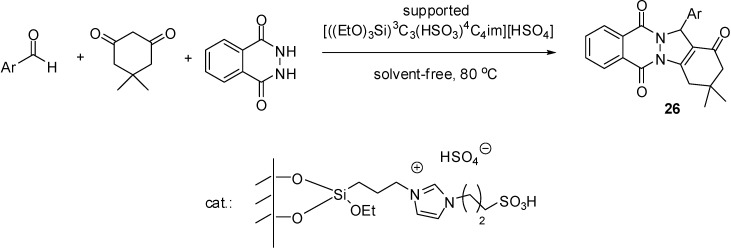
Synthesis of (2H)indazolo[2,1-b]phthalazine-triones (**26**).

An efficient synthesis of (2H)indazolo[2,1-b]phthalazine-triones (**26**, Scheme 38) was performed in the presence of nano-silica supported [((EtO)_3_Si)^3^C_3_(HSO_3_)^4^C_4_im][HSO_4_] [56]. A great variety of aldehydes, including derivatives with electron-donating substituents or electron-withdrawing groups, polycyclic aldehydes and dialdehydes were converted to the products in 77%–96% yields in 10–20 min. The importance of the choice of the support was well demonstrated by the fact that the catalyst supported on Cabosil 20 was more efficient than that obtained with silica gel giving 80% and 94% of the products, respectively, in the reaction of benzaldehyde. This behaviour was attributed to the isolation of active sites on the nano-silica. The catalyst could be used eight times without a significant loss in its activity, giving a yield above 80% even in the 8th run. Comparison of the FT-IR spectra of the catalyst after the 8th run and the fresh catalyst showed no obvious changes in the structure of the catalyst and the characteristic bands. 

**Scheme 39:**
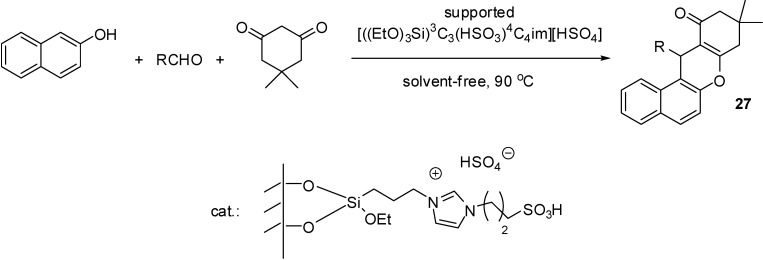
Synthesis of benzoxanthenes (**27**) by a three component condensation.

The same IL ([((EtO)_3_Si)^3^C_3_(HSO_3_)^4^C_4_im][HSO_4_]), supported on silica-coated magnetic nanoparticles was used in a three component condensation leading to benzoxanthenes (**27**, Scheme 39) [57]. The SILP was found to be more active than its unsupported counterpart or another SILP obtained with silica gel as support, resulting in 91%, 76% and 63% yield, respectively. The catalyst could be separated by attaching an external magnet and nearly quantitative catalyst (up to 98%) could be recovered from each run. In a test of six cycles, the catalyst could be reused without any significant loss of catalytic activity. Also, no obvious changes could be observed in the morphology and size or in the IR spectrum of the catalyst after the six runs.

**Scheme 40:**
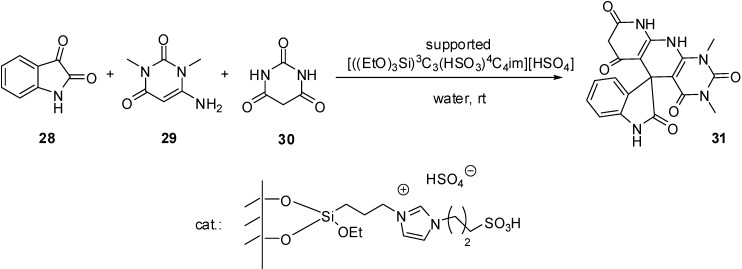
Synthesis of a spirooxindole (**31**) in the presence of [((EtO)_3_Si)^3^C_3_(HSO_3_)^4^C_4_im][HSO4] supported on magnetic nanoparticles.

A very similar catalyst was used in the synthesis of spirooxindole derivatives (e.g., **31**) from isatin (**28**, Scheme 40) or its derivatives, 1,3-dimethyl-2-aminouracil (**29**), and different 1,3-dicarbonyl compounds, e.g., barbituric acid (**30**) [58]. The versatility of the reaction was demonstrated by 24 examples, the products were obtained in 80%–90% yields under really green conditions, *i.e.*, at room temperature in water. By the use of an external magnet, the catalyst could be recovered nearly quantitatively and was reused in 5 cycles with a decrease of the yield from 98% to 87% in the 5th cycle.

### 3.9. Miscellaneous Reactions

Glycerol, one of the renewable resources, could be converted to acrolein, a versatile intermediate, by dehydration using a range of different ILs with alkylsulfonic acid moieties supported on silica [79]. The reactions led to the formation of acrolein, hydroxyacetone and acetaldehyde as major products. The activity of various catalysts prepared using N-containing heterocyclic compounds and phosphines changed in the order: PPh_3_>1-methylimidazole>pyridine~4-picoline~3-picoline~3,4-lutidine>2-picoline. Catalyst coking and degradation of ILs obtained from imidazole, pyridine and 3-picoline was observed, due to the high reaction temperature (275 °C). Besides its good activity, the IL derived from PPh_3_ was found to be stable under the reaction conditions. 

A higher catalytic activity of silica-supported [((EtO)_3_Si)^3^C_3_(HSO_3_)^3^C_3_im]Cl, compared to sulfuric acid or sulfonated silica, was observed in the hydrolysis of cellulose [52]. Hydrolysis was carried out in an IL medium ([C_4_C_1_im]Cl) that readily dissolves cellulose. The superior activity of the SILP catalyst was attributed to hydrogen bonding interactions of the chloride ion with the hydrogens of cellulose OH groups.

The SILP catalyst developed by Chrobok *et al.*, [((EtO)_3_Si)^3^C_3_C_1_im][HSO_4_] supported on a multimodal porous material made from TEOS, was used efficiently in Baeyer–Villiger oxidation [45]. Hydrogen peroxide was proved to be stable in the presence of the acidic catalyst: the concentration of H_2_O_2_ in the test sample, as checked by iodometric titration, did not change. Cyclic ketones were readily oxidised to their corresponding lactones in high yields (60%–91%) under mild conditions within a short time (5–20 h). In cyclopentanone oxidation, the catalyst was reused for three times with no loss of activity, with conversions between 73%–75%. The catalyst could be recovered in 89%–91% yield.

Silica-gel confined acidic ILs, such as [(HO_2_C)C_1_C_1_im]Cl, were used efficiently in the deoximation of cyclohexanone oxime [44]. Almost the same conversions and selectivities were obtained a sin the presence of the pure ILs, but TONs of in the presence of SILPs were almost four times as higher. The 90% conversion and 99% selectivity were maintained when [(HO_2_C)C_1_C_4_im][BF_4_]/silica gel catalyst was reused for the second time, despite a loss of 20% of the original catalyst during separation. Longer reaction time (up to 72 h) was necessary for deoximation of aromatic oximes.

**Scheme 41:**
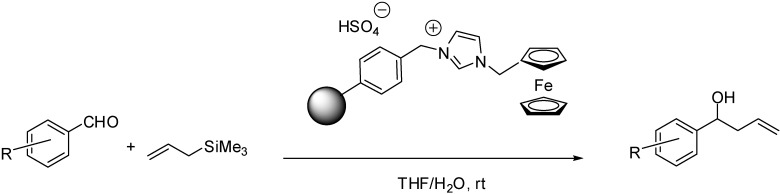
Synthesis of homoallylic alcohols from aromatic aldehydes and allyltrimethylsilane.

Homoallylic alcohols were produced from aromatic aldehydes and allyltrimethylsilane in the presence of a Merrifield resin supported imidazolium IL bearing a pendant ferrocenyl group ([FcC_1_Him][HSO_4_]) (Scheme 41) [63]. The activity of the catalyst was found to remarkably higher (with TONs in the range of 3285–4333 and TOFs in the range of 443–2888 h^−1^) than an analogous SILP without the ferrocenyl moiety ([C_1_Him][HSO_4_]) supported on Merrifield resin, with a TON of 2233 and TOF of 372 h^−1^). These observations were explained by the fact that the presence of ferrocene led to an unsymmetrical charge distribution on the imidazole ring resulting in the significant enhancement of overall micropolarity of the catalyst. Recyclability tests, carried out in the reaction of benzaldehyde and allyltrimethylsilane, proved that the catalyst could be reused with a small loss of activity (with yields of 86% in the 1st run and 78% in the 5th run).

1,4-Conjugate addition of indoles to vinyl ketones were performed in the presence of silica-grafted ILs with sulfonic acid ([(C_1_=C_2_)(HSO_3_)^4^C_4_im][OTf]) or sulfonyl chloride groups ([(C_1_=C_2_)(ClO_2_S)^4^C_4_im][OTf]) [11]. Although the two catalysts showed similar activity, the authors found the latter “much better in terms of general use”, without giving a further explanation. The protocol was applied to various electron-deficient olefins, leading to the products in 67%–93% yields. The catalyst was reused in four cycles, showing a considerable loss of activity. The yield dropped from 87% to 79% in the 4th run, while reaction time was increased from 4 h to 12 h. At the same time, the same catalyst regained its activity after a treatment of thionyl chloride, leading to the product with 90% yield in 2 h.

Lewis acidic chloroaluminate ILs obtained from poly(4-vinylpyridine) or poly(1-vinylimidazole) polymers exhibited similar activity and selectivity to those of their homogeneous counterpart in Diels-Alder reaction of cyclopentadiene with methyl methacrylate [35]. The polymer-supported chloroaluminate catalysts could be recycled five times, but with a 20% loss of initial activity. Atomic absorption measurements of the fresh and used catalysts showed that approximately 6.9 wt. % of the chloroaluminate was lost after the five cycles.

## 4. Conclusions

A great variety of Lewis acidic as well as Brønsted acidic ILs were synthesised and immobilised on different solid supports. The properties of the IL and the support, as well as the immobilisation method have great effects on the morphology, acidity and stability of SILPs. 

All of the above examples demonstrate the efficiency of the SILP concept in acid catalysed reactions. In some cases, the supported catalysts were found to be equally active and selective as the free ILs, but in great majority in the reactions they exerted far better performance. The catalysts could be easily separated and recycling tests were performed during most investigations. With the exception of some catalysts that were found to exert absolutely stable performance, some loss of activity was observed upon reuse in most cases.

Although majority of the research groups carried out careful and detailed investigations of the new materials but there are still a lot of points to be clarified, such as inactivation processes as well as possible routes towards catalyst regeneration. Another less explored point is the use of these catalysts in continuous fix bed reactors that may be the main target of future research.
